# A 104-Ma record of deep-sea Atelostomata (Holasterioda, Spatangoida, irregular echinoids) – a story of persistence, food availability and a big bang

**DOI:** 10.1371/journal.pone.0288046

**Published:** 2023-08-09

**Authors:** Frank Wiese, Nils Schlüter, Jessica Zirkel, Jens O. Herrle, Oliver Friedrich

**Affiliations:** 1 Department of Geobiology, Geoscience Centre, Georg-August-Universität Göttingen, Göttingen, Germany; 2 Institut für Geowissenschaften, Ruprecht-Karls-Universität Heidelberg, Heidelberg, Germany; 3 Museum für Naturkunde, Leibniz Institute for Evolution and Biodiversity Science, Humboldt-Universität zu Berlin, Berlin, Germany; 4 Institute of Geosciences, Goethe-University Frankfurt, Frankfurt, Germany; Università degli Studi di Torino, ITALY

## Abstract

Deep-sea macrobenthic body fossils are scarce due to the lack of deep-sea sedimentary archives in onshore settings. Therefore, hypothesized migrations of shallow shelf taxa into the deep-sea after phases of mass extinction (onshore-offshore pattern in the literature) due to anoxic events is not constrained by the fossil record. To resolve this conundrum, we investigated 1,475 deep-sea sediment samples from the Atlantic, Pacific and Southern oceans (water depth ranging from 200 to 4,700 m), providing 41,460 spine fragments of the crown group Atelostomata (Holasteroida, Spatangoida). We show that the scarce fossil record of deep-sea echinoids is in fact a methodological artefact because it is limited by the almost exclusive use of onshore fossil archives. Our data advocate for a continuous record of deep-sea Atelostomata back to at least 104 Ma (late early Cretaceous), and literature records suggest even an older age (115 Ma). A gradual increase of different spine tip morphologies from the Albian to the Maastrichtian is observed. A subsequent, abrupt reduction in spine size and the loss of morphological inventory in the lowermost Paleogene is interpreted to be an expression of the “Lilliput Effect”, related to nourishment depletion on the sea floor in the course of the Cretaceous-Paleogene (K-Pg) Boundary Event. The recovery from this event lasted at least 5 Ma, and post-K-Pg Boundary Event assemblages progress—without any further morphological breaks—towards the assemblages observed in modern deep-sea environments. Because atelostomate spine morphology is often species-specific, the variations in spine tip morphology trough time would indicate species changes taking place in the deep-sea. This observation is, therefore, interpreted to result from *in-situ* evolution in the deep-sea and not from onshore-offshore migrations. The calculation of the “atelostomate spine accumulation rate” (ASAR) reveals low values in pre-Campanian times, possibly related to high remineralization rates of organic matter in the water column in the course of the mid-Cretaceous Thermal Maximum and its aftermath. A Maastrichtian cooling pulse marks the irreversible onset of fluctuating but generally higher atelostomate biomass that continues throughout the Cenozoic.

## Introduction

The deep-sea, defined as water depths below 200 m, typically represents more than 95% of the oceans. With increasing depth, darkness and water pressures increase, temperatures fall below 4°C, and food availability becomes extremely variable. These features make the world’s largest ecosystem a highly challenging environment. Due to its size and inaccessibility, the bathyal to abyssal plains (below 3,000 m) and the hadal (below 6,000 m) deep-sea trenches are *terrae incognitae*, and only less than 0.0001% of the deep-sea has been explored [1]. Thus, our knowledge of global deep-sea biota, their controlling mechanisms and their evolution is limited, although some census data provide information on regional deep-sea macrofauna [2]. Over the past 20 years, biogeographic patterns [3, 4], latitudinal and depth diversity gradients emerged [5, 6], and a variety of ecological theories converged in principles of deep-sea biodiversity [7–9].

However, concepts of the geological age of the modern deep-sea faunas are ambiguous. The most prominent theory suggests that repeated eradications of deep-sea faunas by Cretaceous Oceanic Anoxic Events (OAEs) and the Paleocene-Eocene Thermal Maximum (PETM, a significant warming of deep-ocean water masses associated with extinctions) [10–14], were succeeded by multiple re-colonisation events of shelf taxa, the so-called “onshore-offshore paradigm” [15]. In this hypothesis it is suggested that the modern deep-sea fauna is younger than the last great oceanographic perturbation, the hyperthermal of the PETM around 56 Ma, which caused widespread disruption of deep-sea benthic communities. However, molecular data from Asselota [16], Isopoda [17], stylasterid corals [18], some fish [19] and fossil data from brittle stars, sea stars, echinoids and holothurians [20, 21] suggest a pre-Cenozoic deep-sea history for these groups. Some taxa even show offshore-onshore trajectories in their history [18, 21]. To date, these antagonistic views have not been tested due to the scarcity of deep-sea sediments and their containing fossil macrofauna onshore. The meagre body fossil record of most of the deep-sea macrobenthos is exemplified by the Spatangoida and Holasteroida (Atelostomata). Since its origin in the early Cretaceous, *ca*. 145 Ma ago [22], this group of irregular echinoids has a continuous fossil record in shelf deposits. However, only seven sites worldwide have provided deep-sea atelostomate body fossils for the Cretaceous–Cenozoic time interval (upper Santonian/lower Campanian of British Columbia, Canada [23], Maastrichtian of northern Spain [24], Campanian to Danian of Italy [25], Miocene of Japan [26], Miocene of Barbados [27], Miocene-Pliocene of Java and Fiji [28], Pliocene of California [29]). However, none of the records exceed palaeo-water depths greater than 2,000 m. Based on this scarce data set, a migration of the Atelostomata into the deep-sea due to increased export productivity was suggested to have taken place at *ca*. 75 Ma (upper Campanian, Upper Cretaceous) [22].

**Fig 1:**
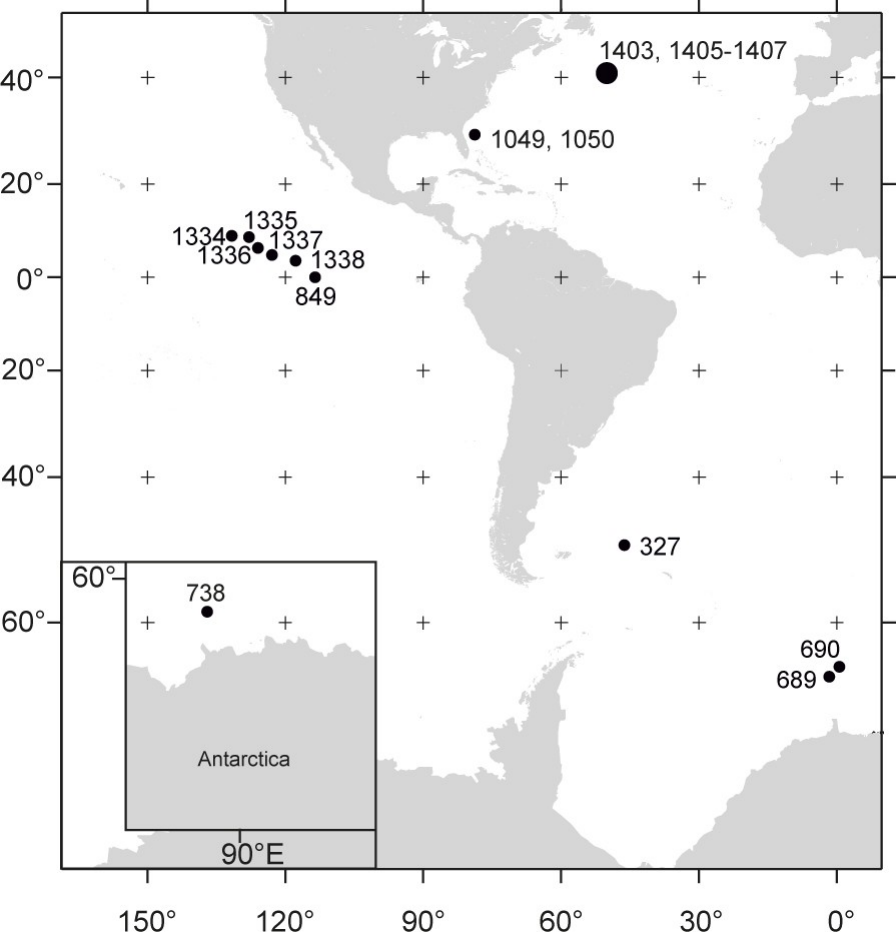
Locations of the presently studied sites (see Table 1 for details).

Here we show that the poor macrofossil record of deep-sea Atelostomata is a methodological artefact and hence provokes a sampling bias: Atelostomata taxonomy is based mainly on the test, disregarding disarticulated material (spines, plates, pedicellariae) [30], and–judging from the scarce literature on atelostomate remains (except of [20, 31])–atelostomate ossicles from deep-sea sediments have traditionally been treated as by-catch. An analysis of more than 1,400 Lower Cretaceous to Pleistocene samples from the Pacific, Atlantic and Southern Oceans (Fig 1 and Table 1)–stratigraphically spanning some critical palaeo-oceanographic intervals such as the OAE 2 (Cenomanian/Turonian boundary), the K-Pg Boundary Event or the Paleocene-Eocene Thermal Maximum, PETM,–produced a large number of atelostomate spines and their fragments.

**Table 1:** Sample information, sample size, stratigraphy, palaeo-water depth and atelostomate spines and fragments recovered. Details on the respective expedition and drillcores, including data on the palaeo-water depths, can be obtained from the homepage of the International Ocean Discovery Program, IODP (https://www.iodp.org/). Colour code for Tabs. 1 and 2: dark green: Lower Cretaceous, light green: Upper Cretaceous, orange: Cenozoic.

stratigraphy	expedition	hole	approx. pwd	sampl.	specim.
Pleistocene	342 Newfoundland	U1407A	3000 m	2	144
Pleistocene	320/321 Tropical Pacific	U1335A	4500 m	1	47
Pleistocene	320/321 Tropical Pacific	U1337A	4700 m	1	26
Pleistocene	320/321 Tropical Pacific	U1338A	3500 m	1	37
Pliocene	138 Eastern Equatorial Pacific	849D	3800 m	41	626
Miocene	320/321 Tropical Pacific	U1336A	4200 m	1	65
Miocene	320/321 Tropical Pacific	U1338B	3600 m	6	126
Miocene	342 Newfoundland	U1405B	3900 m	104	1550
Oligocene	113 Wedell Sea, Maud Rise	689D	2500 m	31	956
Oligocene	320/321 Tropical Pacific	U1334A-C	4050 m	981	25.539
Oligocene	342 Newfoundland	U1406A, B	3800 m	12	95
Eocene	342 Newfoundland	U1407C	2400 m	9	328
Eocene	119 Kerguelen Plateau	738B	1700 m	21	1143
Paleocene	342 Newfoundland	U1407C	2100 m	6	381
Maastrichtian	342 Newfoundland	U1407C	1900 m	2	245
Maastrichtian	342 Newfoundland	U1403B	3500 m	129	350
Maastrichtian	113 Wedell Sea, Maud Rise	690C	1800 m	8	734
Campanian	113 Wedell Sea, Maud Rise	690C	1800 m	50	7170
Campanian	342 Newfoundland	U1407C	1600 m	1	18
Coniac/Santon	342 Newfoundland	U1407C	1000 m	4	52
Turonian	342 Newfoundland	U1407C	500 m	28	521
Cenomanian	342 Newfoundland	U1407C	500 m	30	845
Cenomanian	171B Blake Nose	1050C	2300 m	4	164
Albian	342 Newfoundland	U1407B	200 m	1	250
Albian	342 Newfoundland	U1407C	200 m	1	28
Albian	36 Falkland Plateau	327A	400 m	9	present
Aptian	171B Blake Nose	1049A, C	1500 m	74	present

Based on the hitherto unknown vestiges of fossil deep-sea atelostomate communities down to palaeo-water depths of 4,700 m and literature data, we show the continuous occurrence of this group in the deep-sea for the past *ca*. 115 Ma. In addition to a discussion on the evolution of the Atelostomata in the deep-sea *versus* the onshore-offshore paradigm, we also provide the record of deep-sea macrobenthos across the global K-Pg Boundary Event and its associated extinction horizon. Finally, we expect the calculation of the atelostomate biomass (atelostomate spine accumulation rate: ASAR) for the past 104 Ma to provide an estimate of the relative abundance of deep-sea Atelostomata from the terminal early Cretaceous through the Cenozoic.

## Material and methods

**Fig 2:**
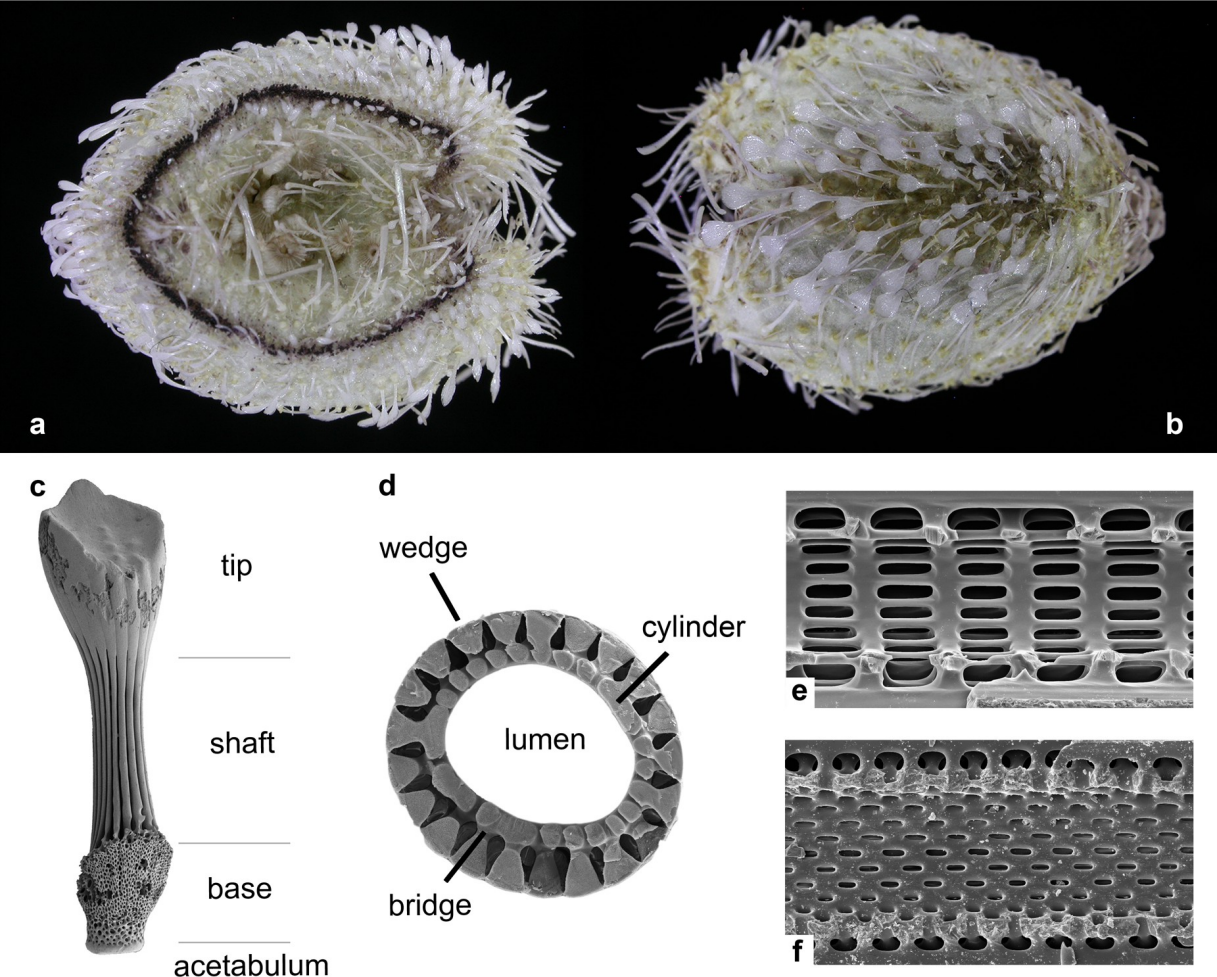
Key morphological features of atelostomate spines. a, b) *Aceste bellidifera* Thomson, 1877 (coast off Liberia, Atlantic), showing the various spine morphologies and size ranges in relation to their functions on the echinoids’ test. a) apical side, b) adapical side (length: *ca*. 15 mm, photo: C. Neumann, Berlin), c-f: morphological features of atelostomate spines. c) general overview (lower Miocene of Newfoundland, Site U1407, unknown species), d) cross section through a spine of *Brissus obesus* Verril, 1867 showing the wedges, bridges and the lumen/cylinder, e) longitudinal section through a spine of *Ceratophysa rosea* (A. Agassiz, 1879), showing the horizontally arranged pores typical of the Holasteroida, f) longitudinal section through a spine of *Eurypatagus parvituberculatus* (Clark, 1924), showing the helicoidal pore orientation in the cylinder, typical of the Spatangoida.

Crown group Atelostomata (Holasteroida, Spatangoida) are detritivore irregular echinoids that originated in the early Cretaceous [25]. Adapted to various functions such as locomotion and burrowing, atelostomate spines and, in particular, their spine tip morphologies, are an expression of functional morphology and often highly variable, depending on their position on the test and their respective functions (see oral/aboral views of *Aceste bellidifera* Thomson, 1877 in Fig 2A and 2B).

**Fig 3:**
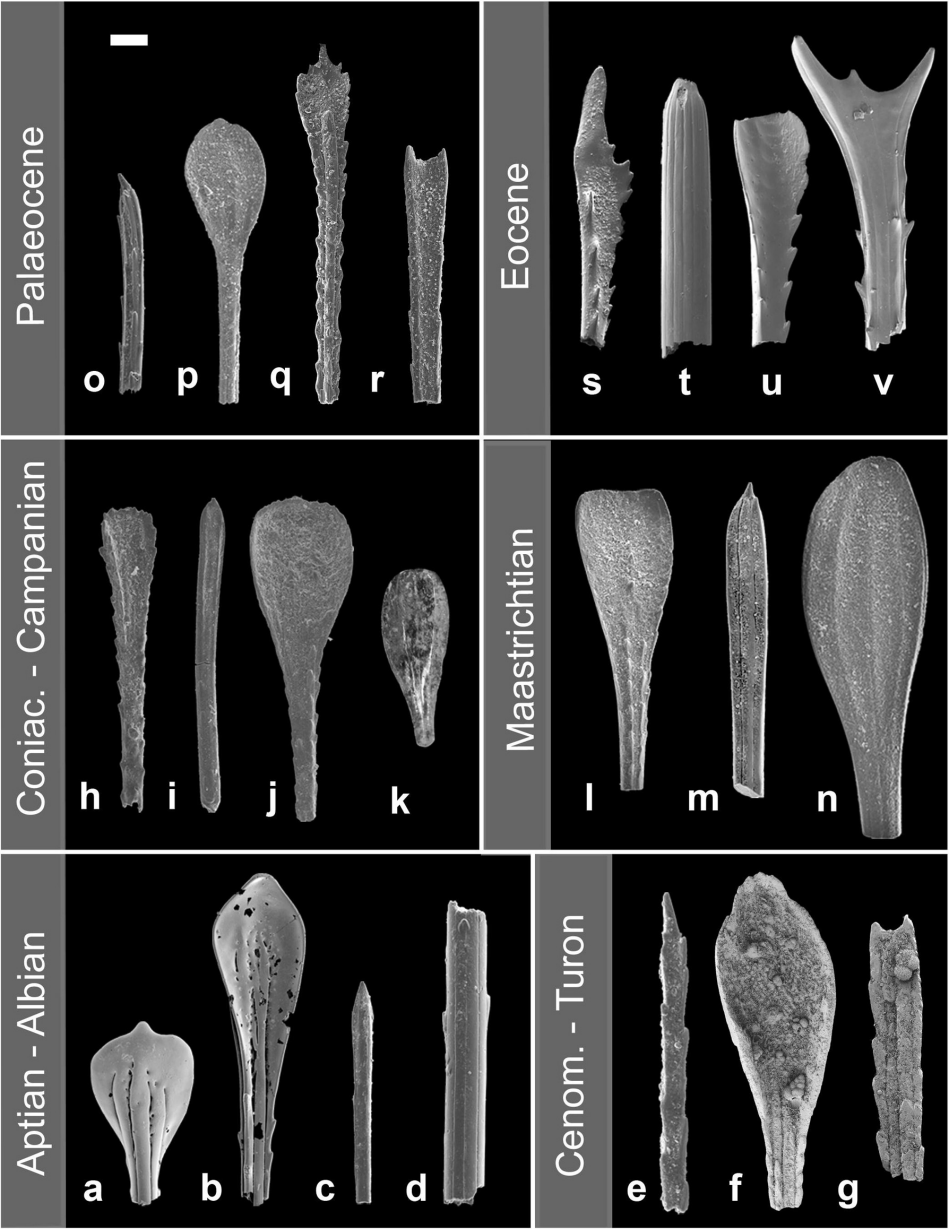
Examples of Aptian to Eocene deep-sea atelostomate spine morphologies. a, b) Sample 1049C-12X-4W, 138–139 cm (Aptian), c, d) Sample U1407C-28X-CCW (Albian), e: Sample U1407C-27X-2W, 50–51 cm (Cenomanian) f, g: Sample U1407A-27X-5A, 60–61 cm (Cenomanian), h-j) Sample U1407C-25H-CCW (Coniacian-Santonian), k) Sample 690C-19X-2W (Campanian), l-n) Sample U1407-C-20H-CC (Maastrichtian), o) Sample U1407C-17H-CC (Paleocene), p, q, r) Sample U1407C-15H-CC (Paleocene), s) Sample U1407C-5H-CC (Eocene), t) Sample U1407C-7H-CC (Eocene), u, v) Sample U1407C-5H-CC (Eocene). Scale bar: *ca*. 100 μm (except n: scale bar 50 μm).

**Fig 4:**
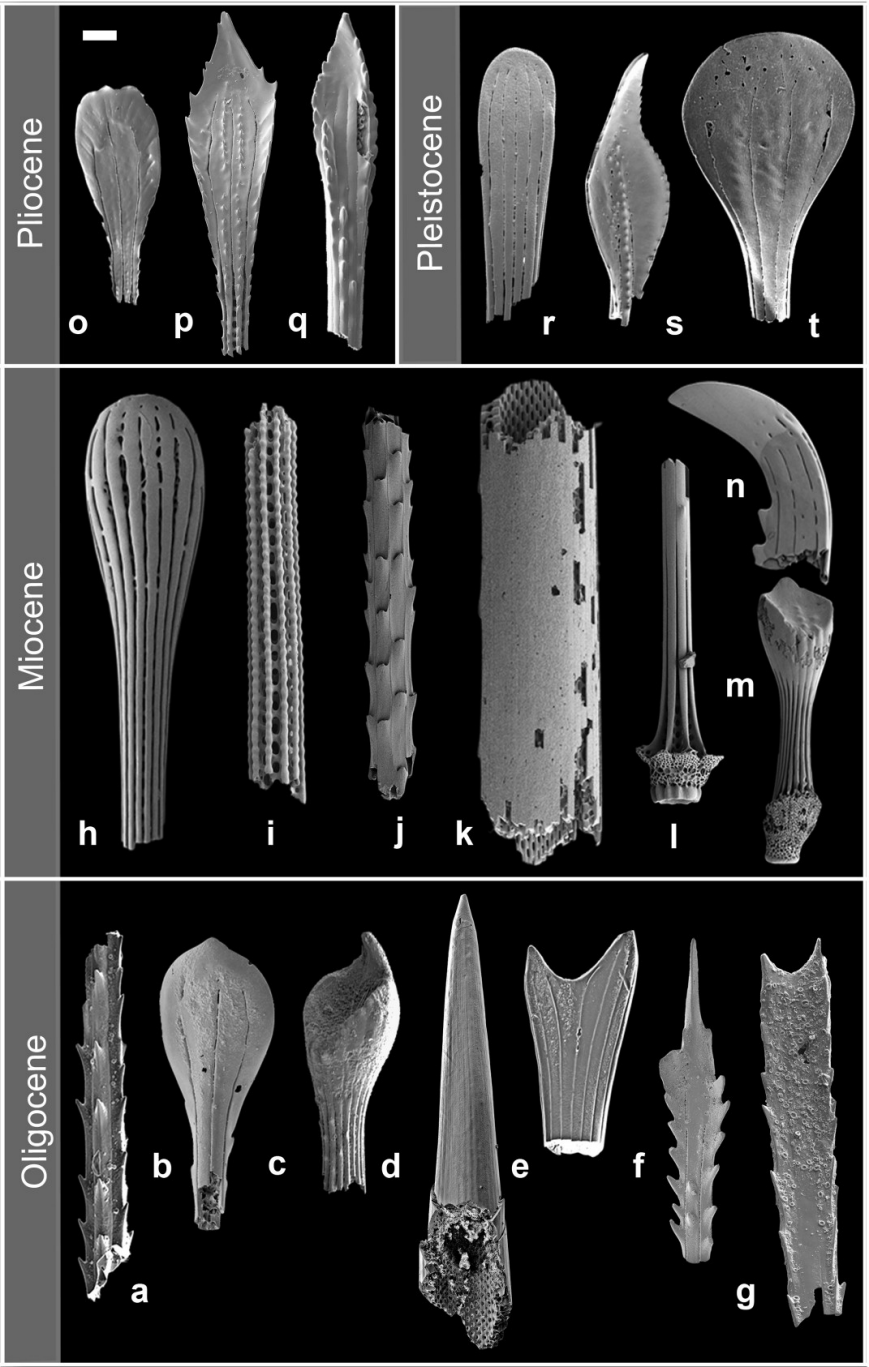
Examples of Oligocene to Pleistocene deep-sea atelostomate spine morphologies. a) Sample U1334C-16H-3W, 148–150 cm (Oligocene), b) Sample U1334C-12H-4W, 43–45 cm (Oligocene), c) 689D-6H-4W, 52–54 cm (Oligocene), d) massive spatangoid spine with acute tip, exposing the spatangoid-like perforation in the cylinder (height: 2.3mm), Sample U1334C-16H-3W, 148–150 cm (Oligocene), e) Sample U1334C-16H-3W, 148–150 cm (Oligocene), f) Sample U1334C-12H-5W, 115–118 cm (Oligocene), g) Sample 689D-6H-4W, 52–54 cm (Oligocene), h-j) Sample U1405B-13H-4W, 40–41 cm (Miocene), k) massive spatangoid spine fragment (length *ca*. 2 mm), Sample U1405B-13H-4W, 40–41 cm (Miocene), l-n) Sample U1405B-13H-4W, 37–38 cm (Miocene), o-q) Sample 849D-8H-2W, 126–128 cm (Pliocene), r-t) Sample U1407A-1H-CC (Pleistocene). Scale bar, if not stated otherwise, *ca*. 100 μm.

**Fig 5:**
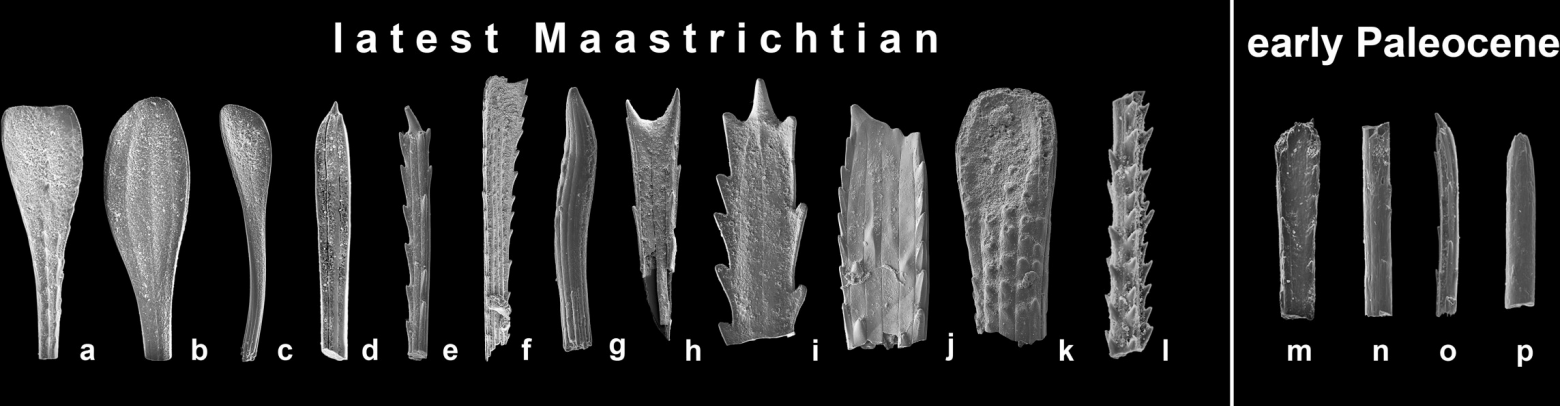
Morphological inventory of atelostomate spine tips and characteristic shaft fragments in the terminal Maastrichtian and early Paleocene, exemplified by Expedition 342 sites U1407 and U1403 (Newfoundland, see Fig 1 and Table 1). Specimens are scaled to similar sizes to illustrate their morphological variability. Actual sizes are given in height (h). a) Sample U1407C-20H-CC (h: 0.88 mm), Maastrichtian, b) Sample U1407C-20H-CC (h: 0.97 mm), Maastrichtian, c) Sample U1407C-20H-CC (h: 1.20 mm), Maastrichtian, d) Sample U1407C-20H-CC (h: 0.80 mm), Maastrichtian, e) Sample U1407C-20H-CC (h: 0.85 mm), Maastrichtian, f) Sample U1403B-28X-2W, 70–72 cm (h: 1.03 mm); g) h: 0.42 mm, h) Sample U1403B-28X-2W, 70–71 cm (h: 0.86 mm), i) Sample U1403B-28X-2W, 76–78 cm (h: 0.55 mm), Maastrichtian, j) Sample U1403B, 28X-2W, 76–78 cm (h: 0.50 mm), Maastrichtian, k) Sample U1407C-20H-CC (h: 0.75 mm), Maastrichtian, l) Sample U1407C-20H-CC (h: 1.09 mm), Maastrichtian, m) Sample U1407C-19H-CC (h: 0.78mm), early Paleocene, n) Sample 1407C-19H-CC (h: 0.69 mm), early Paleocene, o) Sample 1407C-19H-CC (h: 0.54 mm), early Paleocene, p) Sample 1407C-19H-CC (h: 0.55 mm), early Paleocene.

Atelostomate spines can readily be distinguished from spines of other echinoids by their internal microstructure and general morphological characteristics [31]. After the death of the animal and decay of soft tissues, the delicate spines fall off the test and become part of the meso/microfraction of deep-sea sediments. Spines consist of a morphologically variable tip, the shaft and, at their lower end, the base (Fig 2C). In cross-section (Fig 2D), club-shaped wedges form a distinct longitudinal striation, running over the entire length of the shaft. Spines of Atelostomata also reveal a hollow central cavity, the lumen, which is surrounded and enclosed by the cylinder. Their perforation (Fig 2E and 2F) provides a characteristic that distinguishes between the Holasteroida and the Spatangoida (horizontal *versus* helicoidal perforation, respectively) [31]. The ensemble of variable spine morphologies within one taxon (comp. Fig 2A and 2B) can be species-specific [32], why the morphological variability of the spines, in particular the highly variable shape of the spine tips (here referred to as spine tip morphotypes), is an expression of biological species. However, due to the morphological variability of spines within one species, it is currently impossible to relate spine tip morphotypes to a discrete number of biological species, simply because no data exist that relates spines to the test of the animal. While the quantification of spine tip morphotypes is beyond the scope of the present paper, the illustrations in Figs 3–5 may help in grasping the morphological complexity of the studied atelostomate spine tips.

A total of 1,475 deep-sea sediment samples were obtained from Ocean Drilling Program (ODP) and Integrated Ocean Drilling Program/International Ocean Discovery Program (IODP). Lithologically, the sediments reflect typical deep-sea ooze with varying amounts of calcareous nannofossils, foraminifera, diatoms and radiolarians (see respective cruise reports, which can be accessed via https://www.iodp.org/). We analysed all samples quantitatively (Fig 1 and Table 1), i.e., all atelostomate spines and fragments were picked. A palaeo-water depth array of 200 to 4,700 m was realized by the sample set. The material derived mostly from a quarter core 1 cm slice, providing *ca*. 7.5 cm^3^ and sample weights of a few tens of grams. We considered mainly the sizes larger 63 μm, which contains the bulk of atelostomate spines. For Holes U1334A–C, U1406A, B and 849D (Table 2), only the size fraction larger than 125 μm was available for analysis. Photographic documentation was performed with a Scanning Electron Microscope (SEM, *ca*. 1,350 images) at the German universities of Berlin, Frankfurt am Main, Göttingen and Heidelberg.

**Table 2:** Stratigraphic position of samples used to calculate the atelostomate spine accumulation rate (ASAR), including approximate age in Ma, spines per gram sediment (sp/g), the linear sedimentation rate (LSR) and dry bulk density (DBD). For sets of samples, mean values for age and ASAR were calculated, and minima and maxima are given under remarks (comp. also “Material and Methods”). Cen./Turon.: Cenomanian/Turonian boundary interval, Coniac.: Coniacian, Santon.: Santonian, Campan.: Campanian, Maastricht.: Maastrichtian (see “Supplementary Information” for data of sets of samples).

expedition	hole	samples	stage	remarks	age	spines/g	LSR	DBD	ASAR
320/321	U1338A	1H-1W, 0–5	Pleistocene		0.10	2.51	1.5	0.44	1.66
320/321	U1335A	1H-1W, 12–15	Pleistocene		0.30	2.70	0.6	1.38	2.24
320/321	U1337A	1H-CCW, 3–6	Pleistocene		0.44	2.59	1.5	0.60	2.34
342	U1407A	1H-CCc	Pleistocene		1.95	4.30	0.05	1.07	0.23
138	849D	37 samples	Pliocene	mean values (min.: 0.60, max: 6.44)	3.02	1.37	2.85	0.61	2.43
320/321	U1336A	1H-1W, 25–28	Miocene		11.90	3.32	0.90	0.72	2.13
342	U1406A, B	9 samples	Oligocene	mean values (min: 0.35, max: 1.61)	25.07	0.60	2.03	0.79	0.97
320/321	U1334C	981 samples	Oligocene	mean values (min: 0.15, max 48.49)	25.86	1.19	1.88	1.00	2.24
342	U1407C	3H-CC	Eocene		32.65	2.64	0.14	0.85	0.31
342	U1407C	4H-CC	Eocene		35.35	0.95	1.80	0.85	1.46
342	U1407C	5H-CC	Eocene		36.07	0.85	1.80	0.85	1.29
119	738B	21 samples	Eocene	mean values (min: 0.97, max. 11.80)	39.00	3.54	1.18	1.19	4.97
342	U1407C	6H-CC	Eocene		46.23	1.57	2.22	0.89	3.10
342	U1407C	7H-CC	Eocene		46.68	3.41	2.22	0.89	6.74
342	U1407C	8H-CC	Eocene		46.98	1.77	2.22	0.89	3.49
342	U1407C	9H-CC	Eocene		48.06	1.44	0.20	1.04	0.30
342	U1407C	10H-CC	Eocene		49.36	0.44	8.70	1.00	3.86
342	U1407C	11H-CC	Eocene		49.46	2.53	1.04	1.09	2.86
342	U1407C	14H-CC	Paleocene		57.33	4.53	1.04	0.94	4.43
342	U1407C	15H-CC	Paleocene		58.29	1.98	1.04	0.87	1.80
342	U1407C	16H-CC	Paleocene		59.47	2.01	1.04	0.96	2.00
342	U1407C	17H-CC	Paleocene		59.49	1.67	1.04	0.93	1.62
342	U1407C	18H-CC	Paleocene		60.65	0.86	1.04	1.00	0.90
342	U1407C	19H-CC (I)	Paleocene		63.00	7.42	0.27	1.18	2.36
342	U1407C	19H-CC(II)	Paleocene		63.00	12.71	0.27	1.18	4.05
342	U1407C	20H-CC (II)	Maastricht.		69.36	7.11	0.49	1.18	4.11
342	U1407C	20H-CC (I)	Maastricht.		69.36	8.53	0.49	1.18	4.93
342	U1407C	21H-CC (II)	Campan.		76.95	0.69	0.07	1.23	0.06
342	U1407C	21H-CC (I)	Campan.		77.00	0.59	0.07	1.23	0.05
342	U1407C	22H-CC	Santon.		84.00	0.29	0.17	1.44	0.07
342	U1407C	23H-CC	Santon.		85.45	0.37	0.17	1.39	0.09
342	U1407C	24H-CC	Coniac.		87.00	0.17	0.86	1.12	0.16
342	U1407C	25H-CC	Coniac.		88.03	1.11	0.05	1.21	0.07
119	1050C	4 samples	Ce/Turon.	mean values (min: 0.05, max: 0.45)	93.00	0.87	0.14	1.18	0.14
342	U1407A	23 samples	Ce/Turon.	mean values (min: 0.06, max: 3.56)	93.00	0.86	1.16	0.76	0.76
342	U1407C	19 samples	Cenomanian	mean values (min: 0.19, max: 5.16)	94.00	2.07	0.39	1.47	1.19
342	U1407C	26H-CC	Cenomanian		94.65	1.49	0.46	1.26	0.87
342	U1407C	27H-CC	Cenomanian		97.22	0.37	0.36	1.52	0.20
342	U1407C	28H-CC	Cenomanian		100.68	0.39	0.36	1.50	0.21
342	U1407C	29H-CC	Albian		102.08	1.36	0.36	1.52	0.74

Because all spines lack their bases and are mostly fragmented, we measured the minimum spine thickness in 171 samples below (Sample U1407C-20X-CCW) and in 169 samples above (Sample U1407C-19X-CC) the K-Pg Boundary Event of Site 1407 in order to test for possible spine thickness changes across the extinction level. Accordingly, respective mean values were compared, and their significances assessed by performing a Welch t-test. The relative variability of spine thickness is expressed by the coefficient of variation, CV (standard deviation divided by the mean). Statistical analyses were conducted with PAST 4.03 [33] and R v. 4.2.2 [34]. The raw data and the results are compiled in S1 Table.

For 1,128 samples, we calculated the atelostomate spine accumulation rate, ASAR, analogue to the benthic foraminifera accumulation rate [35] as an expression of atelostomate biomass per time (AS: atelostomate spines, DBD: dry bulk density, LSR: linear sedimentation rate):

$$\text{ASAR}\;(\text{spines}/{\text{cm}}^{-2}{\text{kyr}}^{-1})\;=\;\text{AS}/\text{g}\;\times \;\text{DBD}(\text{g}/\text{cm}3)\;\times \;\text{LSR}(\text{cm}/\text{kyr})$$

Where available, the age model, the LSR and the DBD were directly taken or interpolated based on the respective shipboard physical properties data sets. Where DBD was not available or could not be interpolated, it was calculated by using a site-specific equation, which results from a cross plot of wet bulk density, estimated using gamma-ray attenuation porosity evaluator (GRAPE) data sets, against the DBD of selected samples.

For Hole 689D [36]:

$$\text{DBD}\;\left[\text{g}/{\text{cm}}^{-3}\right]=1.58\;\times \;\text{GRAPE}\;-\;1.61$$

For Site U1334 [37]:

$$\text{DBD}=\left[\text{g}/{\text{cm}}^{-3}\right]1.47\;\times \;\text{GRAPE}\;\text{-}\;1.47$$

For Site U1335 [38]:

$$\text{DBD}\;\left[\text{g}/{\text{cm}}^{-3}\right]=\;1.00\;\times \;\text{GRAPE}\;-\;0.02$$

For Site U1336 [39]

$$\text{DBD}\left[\text{g}/{\text{cm}}^{-3}\right]=1.39\;\times \;\text{GRAPE}\;-\;1.37$$

For Site U1337 [40]:

$$\text{DBD}\;\left[\text{g}/{\text{cm}}^{-3}\right]\;=1.6\;\times \;\text{GRAPE}\;-\;1.6$$

For Site U1338 [41]:

$$\text{DBD}=\left[\text{g}/{\text{cm}}^{-3}\right]1.47\;\times \;\text{GRAPE}\;-\;1.47$$

All data used for the calculations are compiled in our Table 2 and in S2–S7 Tables.

## Results

The studied 1,475 samples produced 41,440 atelostomate spines and their fragments. No test fragments were observed. Diagenetic recrystallization of internal spine structures makes it often impossible to assign the spines morphologically to either the Holasteroida or the Spatangoida. Shaft fragments of many specimens were found, but also almost complete spines, lacking their bases or parts of the shaft, occurred frequently (Figs 3 and 4). All observed spines are small, the diameter of the shafts mostly under 100 μm, in thinner variants around 50 μm and even thinner (S1 Table). They fit the size range known for modern deep-sea Holasteroida [42] and specialized deep-sea Spatangoida (Fig 2A and 2B). Shafts are smooth or assembled with thorns, randomly distributed or aligned in rows (Figs 3C, 3O and 3T and 4L), but are generally not very distinct, apart from a few exceptions (Fig 4A and 4I and 4J). A particularly distinct feature is, however, the highly bewildering morphological variability of the spine tip, which shows acute (e.g., Fig 3C and 3E and 3M), spatulate (e.g., Figs 3B, 3N, 3Q and 4C, 4K, 4M), fork-like (Figs 3T and 4D), hoof-like (Fig 4B and 4E), or sickle-shaped (Fig 4G) morphotypes, with serrated (Figs 3Q and 4P) or smooth margins (Figs 3N and 4H), with or without pustulated or tuberculated (Fig 4M and 4S) or perforated (Fig 4P) surfaces. The morphology of the spine tip is already variable in the oldest samples available (Blake Nose, late Aptian/early Albian, *ca*. 115 Ma [20], Fig 3A and 3B and Table 1), and, comparing stage by stage, some spine tip morphologies remain similar (e.g., Fig 3J and 3N and 3P). Other morphotypes are very distinct and restricted to certain time intervals (e.g., Fig 3Q: Paleocene, Fig 3V: Eocene, Fig 4D and 4M: Oligocene–Miocene, Fig 4N: Miocene, Fig 4O–4Q: post-Miocene), but the overall dimensions of the spines consistently remain within an approximate size cluster. Salient are the massive spatangoid spines with undifferentiated, acute tips (Fig 4D and 4K), which, in our samples, do not occur before the Oligocene.

**Fig 6:**
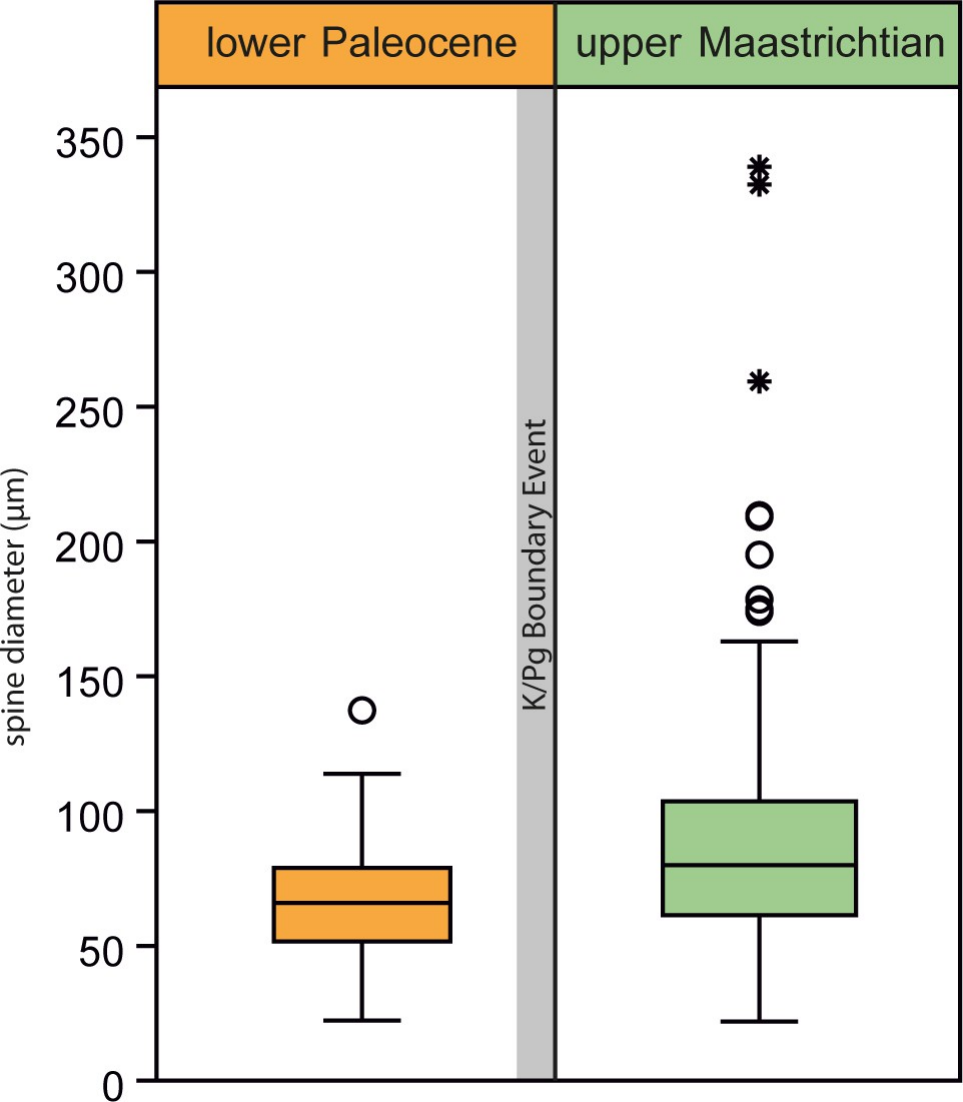
Box plot of atelostomate spine diameters across the K-Pg boundary event (Maastrichtian: Sample U1407-20H-CC, Paleocene: Sample U1407C-19H-CC) (with minima, maxima, median and the 25–75 percent quartiles; see S1 Table). Outliers are shown as circles and stars, respectively; outside the inner fences as circles, beyond the outer fences as stars.

The K-Pg Boundary Event (Hole U1407C) coincides with a remarkable turnover from an assemblage of diversified late Maastrichtian spine tip morphotypes (Fig 5A–5L) towards a less diversified, lowermost Paleocene spine association, consisting mainly of slender, little diverse shaft morphologies (smooth septa, scattered thorns; Fig 5M–5P), and frequent punctuated tips (Fig 5O and 5P) in the 63–125 μm fraction. In the Maastrichtian (Sample U1407C-20H-CC; Table 2), the measurement of spine fragment diameters reveals a mean size of 89.0 μm, with minimum values around 22.0 μm and maximum values reaching 339.0 μm. In the lower Paleocene (Sample U1407C-19H-CC; Table 2), *ca*. 2–3 Ma after the boundary, the mean diameter is 66.3 μm, the minimum is 22.3 μm and the maximum 137.4 μm. The smaller mean value in the Paleogene sample indicates a size decrease of *ca*. 25% compared to the Maastrichtian measurements. The Welch t-test indicates that differences between the two mean values are statistically significant with a *P*-value of 0.0001. The box plot (Fig 6) illustrates the different size distributions before and after the K-Pg Boundary Event, depicting a higher relative variability in the Maastrichtian and a lower relative variability in the lower Paleocene (coefficient of variation, CV Maastrichtian: 50.57, CV Paleogene: 31.62).

The poorly preserved Paleocene spine tip in Fig 5n corresponds in morphology to the Maastrichtian spine tip in Fig 5h, which is the only common element in both time intervals. This lowermost Paleocene association is succeeded by a slow post-event recovery in the aftermath of the extinction level over an interval of at least 5 Ma, showing a continuous shift back towards an increasing number of spine tip morphologies (Fig 4P–4R).

**Fig 7:**
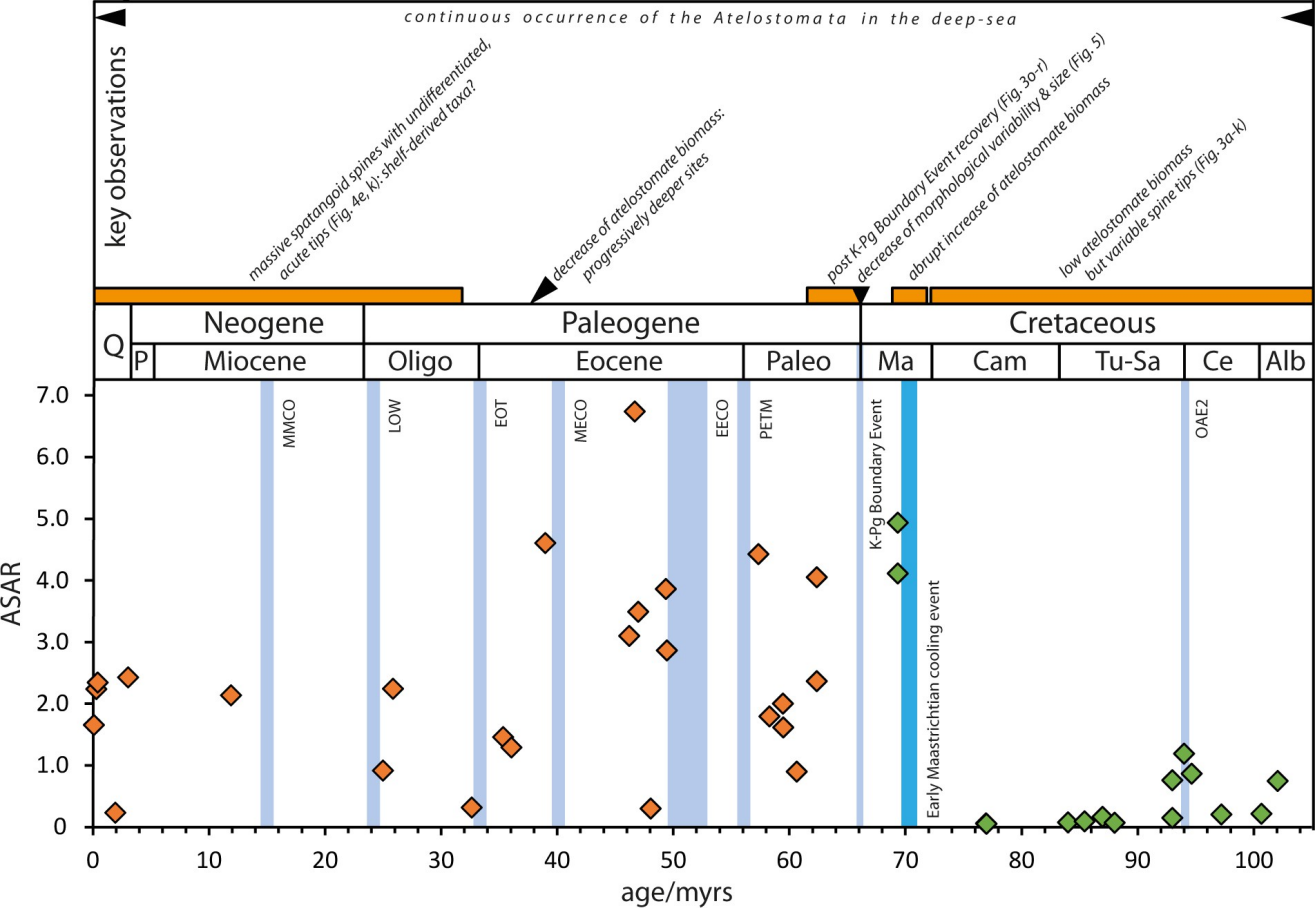
The Atelostomate Spine Accumulation Rate, ASAR (spines/cm^-2^ kyr^-1^), from the terminal early Cretaceous to the Pleistocene (*ca*. 104 Ma) as a proxy for deep-sea Atelostomata biomass. Abbreviations (following [57, 81]): OAE 2: Oceanic Anoxic Event 2, K-Pg Boundary Event: Cretaceous-Paleogene Boundary Event, PETM: Paleocene-Eocene Thermal Maximum, EECO: Early Eocene Climate Optimum, MECO: Middle Eocene Climatic Optimum, EOT: Eocene/Oligocene Transition, LOW: Late Oligocene Warming, MMCO: Mid-Miocene Climatic Optimum.

The ASAR (Fig 7; comp. Table 2) reveals low pre-Maastrichtian values. Upper Cenomanian pre-OAE 2 values at Site U1407 show a single peak value of 2.99, and a set of 19 samples just before OAE 2 comprise a mean value of 1.20. Terminal Cenomanian to Turonian post-OAE 2 values remain low (mean 0.71, peak value 3.52) as do samples from Blake Nose (Site 1050) below and above OAE 2, not exceeding 0.41.The Coniacian-Campanian values remain constantly at an equally low level. Post-Campanian samples show an abrupt increase of the ASAR with values above 4 (Fig 7) and a significant variability of Paleocene to Eocene accumulation rates reaching peak values up to 9.86 (39 Ma, Fig 7). The data points 25.86 Ma (ASAR: 2.24), 25.07 (ASAR: 0.97) and 3.04 Ma (ASAR: 2.13) reflect lowermost ASAR limits, because only the fraction >125 μm was available. Nonetheless, there appears to be a general trend towards lower values in post-Eocene sediments.

## Discussion

Our new record of deep-sea Atelostomata spines since the Albian is the first continuous long-term fossil record of deep-sea macrobenthos published so far. This data set advances the previously patchy and scarce fossil record of deep-sea Atelostomata considerably. Likewise, we present the first fossil record of Atelostomata from deep bathyal to abyssal palaeo-water depths down to 4,700 m. Occurring in all areas considered (Pacific, Atlantic and Southern oceans; Fig 1), a virtually cosmopolitan distribution of this group since the early Cretaceous is likely. The ubiquity of atelostomate spines demonstrates that the group was an integral element of the deep-sea macrofauna since at least 104 Ma. The complete lack of atelostomate test fragments is conspicuous in view of abundant spines. However, the very thin and delicate test of deep-sea Atelostomata is highly vulnerable to physical post-mortem destruction and dissolution (lowered pH by decaying organic matter in the stereom). In addition, reported higher proportions of high-Mg-calcite in the tests compared with a lower Mg-calcite content in the spines [43] suggest selective dissolution of the former. Because the lack of tests was apparent in all our samples, we exclude current-induced sorting.

### A 104 Ma record of deep-sea Atelostomate biomass

Overall, the atelostomate spine accumulation rate, ASAR, as a proxy for the atelostomate biomass accumulation/time (comp. Material and Methods) shows comparatively low pre-Maastrichtian values with peaks hardly reaching 3.5 spines/cm^-2^ kyr^-1^ (Fig 7). Notably, lower values around the OAE 2 from Blake Nose and Newfoundland (Fig 1) demonstrate that this is (at least) a North Atlantic wide phenomenon. The significant increase of post-Campanian ASAR values indicates an increase of atelostomate biomass. However, there is no evidence of increased productivity in the surface water as suggested for this period [22]: some event-like compositional changes among planktonic foraminifera occur [44], and dinoflagellates [45], radiolarians and diatoms reveal no trends, which would indicate increased surface-water productivity in the Maastrichtian. Calcareous nannofossils show even lowered palaeoproductivity towards the Maastrichtian [46]. However, a severe decrease of bottom- and surface-water temperatures occurs towards the Campanian in both hemispheres [47–49], terminated by an abrupt cooling event of both surface and bottom-water temperature around the Campanian–Maastrichtian transition [50–52] (Fig 7). Because there exists a positive correlation between water temperature rise and increased microbial remineralisation rate of particulate organic matter in the water column [53], the Campanian–Maastrichtian cooling event would have reduced the microbial remineralization efficiency of organic matter in the water column significantly. As a result, a higher export of organodetritus as a food source was available in the deep-sea. We suggest that this nourishment boost ignited increasing abundance and biomass of deep-sea Atelostomata as expressed by the increase of the ASAR. To which extent the abrupt temperature decline stimulated a direct physiological response of deep-sea biota [54] in the form of higher biomass or an increase of species number [55] cannot be answered at present. Anecdotal evidence, however, suggests that the higher spine tip variability in the Maastrichtian (Fig 5) at Site U1407C compared to pre-Maastrichtian strata (Fig 3H–3K: total set of spine tip morphotypes) is indicative of a higher species number [32].

Considering reduced re-mineralization rates during cool periods as a trigger for deep-sea atelostomate biomass increase, the Cenomanian/Turonian Cretaceous Thermal Maximum and its aftermath [47, 48, 56] explain the overall lower ASAR values in pre-Maastrichtian times. The post-Eocene decrease of the ASAR (Fig 7) is in an apparent contradiction to the general Cenozoic cooling trend [57]. However, in the case of sites U1334 (25.86 Ma), U1406 (25.07 Ma) and 849 (3.04 Ma) (Fig 7 and Tables 1 and 2), the low ASAR values mark merely the lowermost limits of the atelostomate biomass, because only the fraction >125 μm was available. Nonetheless, these mean values with peaks reaching 48 spines/cm^-2^ kyr^-1^ (Table 2) are still significantly higher than any pre-Maastrichtian mean values. Other post-Eocene sites with a low ASAR reflect increasing water depths (Table 1), associated with a progressive decrease in digestible biomass—as shown by the correlation of primary production, export productivity and zooplankton biomass with depth [58]. This explains overall lower post-Eocene ASAR values, which show, however, still significantly higher peak values than in pre-Maastrichtian times. Because the observed pattern occurs in different ocean basins (Table 2), we exclude facies-related artefacts.

### The K-Pg Boundary Event

The turnover from a variable Maastrichtian spine assemblage towards more slender and less differentiated spines after the K-Pg Boundary Event (Holes U1407C and U1403B, Figs 1 and 5 and Table 1) cannot be quantitatively interpreted in terms of species loss or species turnover (see Material and Methods). The Maastrichtian spines (Fig 5) show a wide range of diameter, including thicker spines and a higher relative variability in diameter as indicated by the CV. This size variability is accompanied by a high morphological variability (Fig 5A–5L). The abundance of more slender spines, less diversified spine types (Fig 5M–5P) and a size decrease of 25%, compared to the Maastrichtian mean value, is sustained 2–3 Ma after the K-Pg Boundary Event and associated with a lower CV (Maastrichtian: 50.57, Paleogene: 31.62). We interpret this signal as a significant perturbation of the deep-sea atelostomate assemblages in conjunction with the K-Pg Boundary Event–an analogue to the contemporaneous size decrease observed in shelf Atelostomata [59]. Smaller deep-sea Atelostomata are in line with food depletion [60–62] and dwarfism (“Lilliput effect”) after the K-Pg Boundary Event, as it is well-recorded from all trophic levels in marine and terrestrial realms, e.g., within planktonic foraminifera [63], calcareous nannoplankton [64], marine molluscs [65], lamniform sharks [66] and in terrestrial trace fossil records [67]. Curiously, the Lilliput effect is also observed in other echinoderms (crinoids) in the aftermath of Palaeozoic extinction events [68]. In Hole U1407C (Fig 1), there is a gap in the sedimentary succession around the boundary interval, and our first sample comes from 560 cm above the gap, i.e., *ca*. 2–3 Ma after the event. During this interval, spines are still smaller and less diversified than in the Maastrichtian (Fig 5M–5P) but occur frequently (max. ASAR: 4.05; Fig 7), supporting a functioning biological pump (which had recovered *ca*. 1.8 Ma after the impact [69]) and the occurrence of an (opportunistic?) post-event atelostomate assemblage. A slight recovery of morphological variability of the spine tips (suggesting a recovery of the atelostomate community) occurs *ca*. 5 Ma after the impact (Fig 3P–3R). These spine morphotypes are morphologically and with respect to size in part reminiscent of Maastrichtian morphotypes (e.g., Fig 3P), suggesting the recurrence of pre-K-Pg Boundary Event conditions. Our observations are, therefore, in line with the general faunal patterns [69] and the knowledge that deep-sea macrobenthos is intimately linked to surface-water processes and changes in export productivity, making deep-sea biota highly vulnerable to global change [70]. Furthermore, our data demonstrate that a resilience of deep-sea biota against major extinction events, as it has been suggested in previous studies [20], does not exist.

### No onshore-offshore trajectories?

Originally deduced from distal and proximal shelf community distribution patterns [15, 71], the onshore-offshore paradigm was progressively applied to explain the origin of the modern deep-sea fauna, particularly in the context of the hypothesis that repeated eradication of deep-sea communities by Cretaceous OAEs was followed by the successive resettlement of the deep-sea by shelf-derived taxa [11]. However, this hypothesis has eroded over the last decades, because the antiquity of numerous recent deep-sea clades [16–19, 72, 73] and entire faunal assemblages was shown [20, 74], inclusive seep communities [75], questioning the general validity of the onshore-offshore paradigm as an explanation for the origin of the modern deep-sea fauna [20, 74]. Our results are in line with the above conclusions, and we show–in contrast to previous suggestions [22]–that the Atelostomata inhabited the deep-sea permanently at least since 104 Ma. Previous studies date their occurrence in the deep-sea even back into the upper Aptian (*ca*. 115 Ma) [20, 31]. Because the spine morphologies are often species-specific [32], the manifold spine tip morphotypes (Figs 3–5) are indicative of a number of hitherto unknown deep-sea atelostomate taxa. The temporal succession of highly specialized spine tips–partly morphologically unique and stratigraphically limited (e.g., Figs 3Q, 3S, 3V and 4F, 4I, 4N, 4P and 5F, 5I, 5K) concludes evolving deep-sea Atelostomata assemblages. We have not been able to detect major morphological breaks among the spine assemblages, which would point towards an immigration of shelf taxa into the deep-sea, likely revealed by abrupt changes in spine morphotype composition, deviant phenotypes or spine sizes.

Considering the pre-Jurassic history of parts of the modern deep-sea macrobenthos [72–74, 76], benthic deep-sea ecosystems had tens of millions of years of time to diversify and stabilize to climax communities. There is no reason to assume less diversified deep-sea ecosystems with lower diversities compared to the modern deep-sea benthos [77]. Any inferred multiple invasion attempts by the end of the Cretaceous and later [22, 24, 78] would not have encountered an empty space, but would have come across a stable climax community, providing solid ecological resistance [79]. In this context, we hypothesise that the observed morphological variability of atelostomate spines is expression of *in situ* evolution in the deep-sea. We also suggest that the long post-event recovery phase of at least 5 Ma after the K-Pg Boundary Event, exhibiting gradually more differentiated Cenozoic spine tip morphotypes, is an intrinsic deep-sea process and not caused by onshore-offshore migrations. However, sporadic immigration of shelf taxa into the deep-sea, e.g., via isothermal water masses [80], cannot be excluded, and the scarce occurrences of Maastrichtian Atelostomata in distal shelf or slope settings [22, 78] may reflect such immigration attempts. Likewise, the massive Oligocene/Miocene spatangoid spines (Fig 4D and 4K) could be an expression of such an event. Nonetheless, offshore-onshore migrations known from other groups [18, 21] can also not be completely excluded for the Atelostomata, and the occurrence of the holasteroid *Galeaster* (the precursor of the exclusive deep-sea Pourtalesiidae [78]) in slope settings could be interpreted as such.

## Conclusions

The use of deep-sea sediment samples opens an entirely new pathway towards an unexplored fossil archive of an evolved group of deuterostome deep-sea macrobenthos, the Atelostomata. This enables us, for the first time, to gain a comprehensive long-term deep-sea macrobenthos fossil record from the late early Cretaceous to the Pleistocene for this group. Our new data sets represent the first fossil record of atelostomate echinoids from depths larger than 2,000 m to 4,700 m, and the first long-term fossil record of deep-sea macrobenthos in general. Our main results are summarized as follows.

- The Atelostomata persist in the deep-sea at least since the late Aptian/early Albian (*ca*. 115 Ma), and our 104 Ma atelostomate record from the late Albian on is the first continuous deep-sea fossil record of benthic macroinvertebrates down to palaeo-water depths of 4,700 m.
- The 104 Ma record of atelostomate biomass (ASAR) shows constantly lower values in pre-Maastrichtian times compared to the younger record. An abrupt increase in post-Campanian sediments is inferred to be related to the Campanian/Maastrichtian cooling event, which acted as a booster for atelostomate evolution. Since then, mean atelostomate biomass remained irreversibly at a higher level.
- Processes associated with the K-Pg Boundary Event severely affected the deep-sea Atelostomata. More slender and less diversified spine tip morphotypes paralleled by a size decrease of around 25% were sustained still 2–3 Ma after the event (“Lilliput Effect”), succeeded by a gradual recovery of spine tip variability over at least 5–6 Ma.
- Continuous changes of species-specific spine tip morphotypes from the upper Aptian/lower Albian to the Maastrichtian and following the K-Pg Boundary Event are indicative of an *in situ* evolution within deep-sea Atelostomata.
- No evidence of onshore-offshore trajectories (i.e., the migration of shelf taxa into the deep-sea) can be found.

Due to the frequent occurrence of atelostomate spines in deep-sea sediments and a continuous stratigraphic record since the late early Cretaceous, we advocate the use of atelostomate spines to decipher the response of fossil deep-sea macrobenthos to critical palaeo-climatological intervals. Applying the ASAR and treating spine tip morphotypes as parataxonomic units, mass accumulation rates and diversity can quantitatively be studied across global warming episodes, such as, e.g., the Mid-Miocene climate optimum [81] or Cenozoic hyperthermal events [57]. Documentation of macrobenthos faunal change can help to decipher the effects of global warming and its associated changes of export productivity on deep-sea macrobenthos diversity, guild structures and biomass in large parts of the oceans [82]. This makes the Atelostomata the only potential macrobenthos model taxon providing the historical component needed to predict the effects of ongoing global warming on deep-sea macrobenthos.

## Supporting information

S1 Table Data set spine diameter across the K-Pg Boundary (Fig 6). (PDF)

S2 Table Data set to calculate the Atelostomate Spine Accumulation Rate, ASAR, Hole 738B. (PDF)

S3 Table Data set to calculate the Atelostomate Spine Accumulation Rate, ASAR, Hole 849D. (PDF)

S4 Table Data set to calculate the Atelostomate Spine Accumulation Rate, ASAR, Hole 1050C. (PDF)

S5 Table Data set to calculate the Atelostomate Spine Accumulation Rate, ASAR, Site U1334. (PDF)

S6 Table Data set to calculate the Atelostomate Spine Accumulation Rate, ASAR, Holes U1406 A, B. (PDF)

S7 Table Data set to calculate the Atelostomate Spine Accumulation Rate, ASAR, Holes U1407A, C. (PDF)
