# Supplementary material for: A 104-Ma record of deep-sea Atelostomata (Holasterioda, Spatangoida, irregular echinoids) – a story of persistence, food availability and a big bang
Source: PLoS One. 2023 Aug 9;18(8):e0288046. doi: 10.1371/journal.pone.0288046 (PMC10411753; doi:10.1371/journal.pone.0288046)
Supplement: S1 Table — (PDF) [file pone.0288046.s001.pdf]

Spine diameter across the K-Pg Bounday Event (all values in µm)

No.	Lower Paleocene U1407C-19H-CC	Upper Maastrichtian U1407C-20H-CC
1	81.66	75.35
2	96.54	115.05
3	49.91	137.64
4	81.32	102.63
5	91.01	21.96
6	44.50	62.81
7	95.32	106.61
8	61.38	98.37
9	72.06	83.98
10	96.62	61.38
11	75.75	82.79
12	83.39	91.17
13	88.53	42.69
14	106.40	104.59
15	42.48	51.23
16	29.32	58.65
17	66.22	69.71
18	36.17	54.74
19	76.65	54.84
20	64.97	80.45
21	64.36	109.48
22	72.21	37.50
23	38.15	50.62
24	62.98	93.98
25	88.53	162.96
26	53.48	108.77
27	54.84	51.23
28	54.74	59.82
29	99.96	64.04
30	70.08	131.04
31	45.97	47.73
32	65.91	124.14
33	58.36	76.82
34	53.26	90.87
35	101.06	115.01
36	75.70	59.44
37	75.15	68.21
38	43.62	92.15
39	77.93	95.70
40	100.28	62.41
41	53.68	134.49
42	51.40	49.72
43	56.99	84.32
44	51.38	66.91
45	57.25	97.35
46	62.81	98.68

	U1407C-19H-CC	U1407C-20H-CC
**Min**	22.32	21.96
**Max**	137.38	339.00
**Mean**	66.26	89.87
**Median**	65.91	79.97
**25 percentil**	51.61	61.38
**75 percentil**	78.97	103.64
**CV**	31.62	50.57

CV: coefficient of variation

47	55.56	54.32
48	42.61	62.68
49	76.09	85.93
50	55.75	73.24
51	70.99	74.50
52	71.66	55.18
53	65.37	32.07
54	76.84	73.25
55	82.91	93.56
56	24.44	209.77
57	57.27	130.60
58	42.61	84.27
59	53.06	97.75
60	51.81	110.17
61	44.26	116.02
62	46.78	71.46
63	78.88	100.09
64	36.73	72.99
65	49.72	118.54
66	47.23	117.57
67	37.69	123.88
68	22.68	51.40
69	31.65	65.57
70	39.95	109.24
71	69.20	116.12
72	70.92	99.87
73	44.08	100.86
74	85.40	87.57
75	55.00	99.26
76	72.74	39.72
77	48.47	56.07
78	102.08	173.90
79	79.05	90.12
80	50.67	73.72
81	68.23	70.77
82	52.32	58.36
83	52.22	54.40
84	47.28	64.04
85	55.13	58.98
86	70.89	100.47
87	59.55	69.87
88	54.59	75.91
89	55.94	104.18
90	41.13	66.34
91	23.56	174.86
92	57.64	151.59
93	98.87	60.74
94	49.91	64.54
95	42.63	146.25
96	60.99	259.41

97	99.38	86.64
98	68.74	45.27
99	85.60	40.74
100	101.49	67.02
101	65.50	84.21
102	92.84	60.79
103	45.27	68.26
104	58.24	92.49
105	77.24	73.96
106	113.84	82.78
107	66.86	48.96
108	54.66	62.58
109	50.88	53.03
110	101.23	51.16
111	60.22	45.91
112	45.06	89.45
113	77.64	47.71
114	88.86	80.18
115	102.53	69.45
116	53.28	117.55
117	68.33	93.72
118	66.63	58.65
119	89.46	109.49
120	22.32	56.77
121	66.22	58.98
122	59.44	65.01
123	73.44	113.99
124	25.85	64.36
125	66.58	78.84
126	69.45	101.20
127	73.96	97.28
128	36.83	81.92
129	43.21	50.97
130	73.53	53.26
131	77.23	103.18
132	70.79	195.09
133	67.37	209.19
134	109.80	103.86
135	72.76	106.13
136	80.44	209.14
137	37.14	95.66
138	42.16	49.54
139	77.93	76.50
140	56.52	117.18
141	68.03	97.78
142	93.66	59.62
143	82.35	178.53
144	47.64	56.62
145	59.98	55.24
146	55.18	81.22

147	73.27	74.11
148	101.20	61.83
149	67.17	73.66
150	87.88	77.57
151	59.55	339.00
152	42.63	66.53
153	105.76	72.85
154	52.20	63.04
155	137.38	83.53
156	86.64	68.47
157	39.95	79.43
158	94.69	108.98
159	93.13	92.41
160	102.66	79.97
161	62.22	59.36
162	66.75	92.19
163	78.45	58.78
164	93.13	103.64
165	64.35	67.18
166	68.08	332.44
167	54.23	162.70
168	82.28	130.43
169	79.09	70.21
170		87.27
171		123.69
