# Supplementary material for: A 104-Ma record of deep-sea Atelostomata (Holasterioda, Spatangoida, irregular echinoids) – a story of persistence, food availability and a big bang
Source: PLoS One. 2023 Aug 9;18(8):e0288046. doi: 10.1371/journal.pone.0288046 (PMC10411753; doi:10.1371/journal.pone.0288046)
Supplement: S2 Table — (PDF) [file pone.0288046.s002.pdf]

data set Hole 738B

sample id	hole	age in Fig. 7	spines	spines/g	dry weight (g)	LSR	DBD	ASAR
1	738B	39.00	40	2.34	17.13	1.18	1.19	3.28
2	738B	39.00	13	1.12	11.64	1.18	1.19	1.57
3	738B	39.00	22	1.54	14.32	1.18	1.19	2.16
4	738B	39.00	18	1.02	17.64	1.18	1.19	1.43
5	738B	39.00	20	1.47	13.56	1.18	1.19	2.07
6	738B	39.00	17	1.68	10.14	1.18	1.19	2.35
7	738B	39.00	40	2.22	18.02	1.18	1.19	3.12
8	738B	39.00	35	2.78	12.61	1.18	1.19	3.90
9	738B	39.00	32	2.13	15.01	1.18	1.19	2.99
10	738B	39.00	63	7.02	8.97	1.18	1.19	9.86
11	738B	39.00	42	2.96	14.2	1.18	1.19	4.15
12	738B	39.00	37	3.56	10.4	1.18	1.19	5.00
13	738B	39.00	130	8.25	15.76	1.18	1.19	11.58
14	738B	39.00	112	7.04	15.9	1.18	1.19	9.89
15	738B	39.00	176	8.40	20.95	1.18	1.19	11.80
16	738B	39.00	130	7.72	16.84	1.18	1.19	10.84
17	738B	39.00	43	2.52	17.03	1.18	1.19	3.55
18	738B	39.00	98	5.88	16.66	1.18	1.19	8.26
19	738B	39.00	34	2.19	15.51	1.18	1.19	3.08
20	738B	39.00	9	0.69	13.02	1.18	1.19	0.97
21	738B	39.00	32	1.88	17.04	1.18	1.11	2.46

LSR: linear sedimentation rate
DBD: dry bulk density
ASAR: atelostomate spine accumulation rate
