# Supplementary material for: A 104-Ma record of deep-sea Atelostomata (Holasterioda, Spatangoida, irregular echinoids) – a story of persistence, food availability and a big bang
Source: PLoS One. 2023 Aug 9;18(8):e0288046. doi: 10.1371/journal.pone.0288046 (PMC10411753; doi:10.1371/journal.pone.0288046)
Supplement: S3 Table — (PDF) [file pone.0288046.s003.pdf]

data set Hole 849D

sample id	hole	core	age in Fig. 7	spines	spines/g	dry weight (g)	LSR	DBD	ASAR
1	849D	8H-2W	3.02myr	16	1.34	11.92	2.95	0.82	3.25
2	849D	8H-2W	3.02myr	11	1.00	11.05	2.95	0.71	2.08
3	849D	8H-2W	3.02myr	17	1.36	12.54	2.95	0.71	2.82
4	849D	8H-2W	3.02myr	25	1.96	12.77	2.95	0.67	3.89
5	849D	8H-2W	3.02myr	13	0.99	13.07	2.95	0.74	2.17
6	849D	8H-2W	3.02myr	10	0.82	12.18	2.88	0.72	1.71
7	849D	8H-2W	3.02myr	17	2.48	6.85	2.88	0.75	5.35
8	849D	8H-2W	3.02myr	22	1.72	12.81	2.88	0.73	3.61
9	849D	8H-2W	3.02myr	24	2.03	11.84	2.88	0.68	3.97
10	849D	8H-2W	3.02myr	23	1.97	11.7	2.88	0.68	3.85
11	849D	8H-2W	3.02myr	10	0.79	12.64	2.88	0.66	1.52
12	849D	8H-2W	3.02myr	6	0.58	10.26	2.88	0.67	1.13
13	849D	8H-2W	3.02myr	11	0.93	11.8	2.88	0.58	1.57
14	849D	8H-2W	3.02myr	5	0.58	8.59	2.88	0.50	0.84
15	849D	8H-2W	3.02myr	6	0.76	7.85	2.88	0.51	1.11
16	849D	8H-2W	3.02myr	14	1.44	9.74	2.88	0.61	2.53
17	849D	8H-2W	3.02myr	10	1.05	9.55	2.88	0.55	1.66
18	849D	8H-2W	3.02myr	8	0.87	9.17	2.88	0.51	1.29
19	849D	8H-2W	3.02myr	12	1.28	9.35	2.88	0.50	1.86
20	849D	8H-2W	3.02myr	9	1.25	7.21	2.88	0.48	1.73
21	849D	8H-2W	3.02myr	16	1.93	8.27	2.88	0.40	2.23
22	849D	8H-2W	3.02myr	7	0.88	7.94	2.88	0.48	1.23
23	849D	8H-2W	3.02myr	9	1.04	8.64	2.88	0.49	1.47
24	849D	8H-2W	3.02myr	5	0.46	10.93	2.88	0.46	0.60
25	849D	8H-2W	3.02myr	16	1.52	10.55	2.88	0.54	2.36
26	849D	8H-2W	3.02myr	13	1.38	9.41	2.88	0.57	2.26
27	849D	8H-2W	3.02myr	14	1.65	8.51	2.88	0.57	2.72
28	849D	8H-2W	3.02myr	18	1.84	9.77	2.88	0.58	3.07
29	849D	8H-2W	3.02myr	17	1.82	9.33	2.88	0.57	2.98
30	849D	8H-2W	3.02myr	12	1.37	8.73	2.88	0.53	2.10
31	849D	8H-2W	3.02myr	14	1.59	8.78	2.88	0.65	3.01
32	849D	8H-2W	3.02myr	33	2.99	11.02	2.88	0.75	6.44
33	849D	8H-2W	3.02myr	25	2.02	12.37	2.88	0.71	4.14
34	849D	8H-2W	3.02myr	20	1.62	12.38	2.52	0.66	2.70
35	849D	8H-2W	3.02myr	14	1.24	11.3	2.52	0.64	2.01
36	849D	8H-2W	3.02myr	18	1.47	12.25	2.52	0.63	2.35
37	849D	8H-2W	3.02myr	10	0.78	12.74	2.52	0.71	1.40

LSR: linear sedimentation rate
DBD: dry bulk density
ASAR: atelostomate spine accumulation rate
