# Supplementary material for: A 104-Ma record of deep-sea Atelostomata (Holasterioda, Spatangoida, irregular echinoids) – a story of persistence, food availability and a big bang
Source: PLoS One. 2023 Aug 9;18(8):e0288046. doi: 10.1371/journal.pone.0288046 (PMC10411753; doi:10.1371/journal.pone.0288046)
Supplement: S4 Table — (PDF) [file pone.0288046.s004.pdf]

data set Hole 1050C

sample id	hole	core	age in Fig. 7	spines	spines/g	dry weight (g)	LSR	DBD	ASAR
1	1050C	21R-1W	93.00	46	0.93	49.5	0.14	1.8	0.23
2	1050C	21R-1W	93.00	82	1.80	45.5	0.14	1.8	0.45
3	1050C	21R-1W	93.00	9	0.21	43.2	0.14	1.8	0.05
4	1050C	21R-1W	93.00	27	0.56	48.6	0.14	1.8	0.14

LSR: linear sedimentation rate
DBD: dry bulk density
ASAR: atelostomate spine accumulation rate
