# Supplementary material for: A 104-Ma record of deep-sea Atelostomata (Holasterioda, Spatangoida, irregular echinoids) – a story of persistence, food availability and a big bang
Source: PLoS One. 2023 Aug 9;18(8):e0288046. doi: 10.1371/journal.pone.0288046 (PMC10411753; doi:10.1371/journal.pone.0288046)
Supplement: S5 Table — (PDF) [file pone.0288046.s005.pdf]

sample id	hole	age in Fig. 7	spines	spines/g	dry weight (g)	LSR	DBD	ASAR
**1**	U1334A	25.86	3	0.22	13.66	2.20	0.90	0.43
**2**	U1334A	25.86	4	0.31	12.82	2.20	0.89	0.61
**3**	U1334A	25.86	10	0.73	13.68	2.20	0.92	1.48
**4**	U1334A	25.86	4	0.39	10.27	2.20	0.96	0.82
**5**	U1334A	25.86	8	0.60	13.24	2.20	0.94	1.25
**6**	U1334A	25.86	8	0.66	12.16	2.20	0.98	1.42
**7**	U1334A	25.86	8	0.69	11.65	2.20	0.95	1.44
**8**	U1334A	25.86	6	0.44	13.59	2.20	0.90	0.88
**9**	U1334A	25.86	4	0.28	14.39	2.20	0.90	0.55
**10**	U1334A	25.86	2	0.17	12.05	2.20	0.88	0.32
**11**	U1334A	25.86	21	1.50	13.96	2.20	0.93	3.08
**12**	U1334A	25.86	13	1.03	12.6	2.20	0.94	2.14
**13**	U1334A	25.86	11	0.95	11.59	2.20	0.91	1.89
**14**	U1334A	25.86	5	0.39	12.92	2.20	0.99	0.84
**15**	U1334A	25.86	11	0.80	13.69	2.20	0.95	1.68
**16**	U1334A	25.86	23	1.52	15.1	2.20	0.96	3.22
**17**	U1334A	25.86	16	1.39	11.51	2.20	0.99	3.02
**18**	U1334A	25.86	17	1.29	13.18	2.20	1.01	2.87
**19**	U1334A	25.86	35	2.24	15.61	0.88	1.02	2.00
**20**	U1334A	25.86	11	0.86	12.78	0.88	0.98	0.74
**21**	U1334A	25.86	11	0.90	12.22	0.88	1.01	0.80
**22**	U1334A	25.86	3	0.24	12.42	0.88	1.00	0.21
**23**	U1334A	25.86	4	0.34	11.66	0.88	0.96	0.29
**24**	U1334A	25.86	7	0.49	14.41	0.88	1.01	0.43
**25**	U1334A	25.86	6	0.46	13.09	1.20	1.01	0.56
**26**	U1334A	25.86	7	0.66	10.67	1.20	0.95	0.75
**27**	U1334A	25.86	7	0.54	12.9	1.20	0.96	0.63
**28**	U1334A	25.86	5	0.37	13.49	1.20	0.94	0.42
**29**	U1334A	25.86	7	0.56	12.41	1.20	0.90	0.61
**30**	U1334A	25.86	9	0.77	11.7	1.20	0.96	0.89
**31**	U1334A	25.86	35	2.83	12.35	1.20	0.96	3.28
**32**	U1334A	25.86	28	2.16	12.96	1.20	0.96	2.49
**33**	U1334A	25.86	25	2.00	12.47	1.20	0.95	2.30
**34**	U1334B	25.86	11	1.50	7.35	1.20	0.94	1.69
**35**	U1334B	25.86	11	1.42	7.76	1.20	0.97	1.65
**36**	U1334B	25.86	14	1.59	8.82	1.20	0.95	1.81
**37**	U1334B	25.86	13	1.46	8.92	1.20	0.96	1.69
**38**	U1334B	25.86	5	0.59	8.51	1.02	0.94	0.56
**39**	U1334B	25.86	9	1.18	7.62	1.02	0.89	1.07
**40**	U1334B	25.86	9	1.16	7.78	1.02	0.84	0.99
**41**	U1334B	25.86	13	1.64	7.95	1.02	0.88	1.46
**42**	U1334B	25.86	4	0.54	7.36	1.02	0.86	0.47
**43**	U1334B	25.86	2	0.28	7.04	1.02	0.85	0.24
**44**	U1334B	25.86	3	0.35	8.63	1.02	0.88	0.31
**45**	U1334B	25.86	4	0.55	7.29	1.02	0.84	0.47

**46**	U1334B	25.86	11	1.36	8.07	1.02	0.84	1.16
**47**	U1334B	25.86	15	1.95	7.7	1.02	0.85	1.69
**48**	U1334B	25.86	8	1.12	7.13	1.02	0.86	0.98
**49**	U1334B	25.86	12	1.72	6.98	1.41	0.84	2.04
**50**	U1334B	25.86	7	1.14	6.13	1.41	0.85	1.37
**51**	U1334B	25.86	10	1.32	7.6	1.41	0.86	1.60
**52**	U1334B	25.86	11	1.54	7.14	1.41	0.83	1.80
**53**	U1334B	25.86	10	1.47	6.81	1.41	0.77	1.60
**54**	U1334B	25.86	11	1.63	6.73	1.41	0.76	1.74
**55**	U1334B	25.86	9	1.35	6.68	1.41	0.76	1.44
**56**	U1334B	25.86	1	0.14	7.15	1.41	0.78	0.15
**57**	U1334B	25.86	10	1.25	7.97	1.41	0.78	1.38
**58**	U1334B	25.86	9	1.18	7.62	1.41	0.83	1.38
**59**	U1334B	25.86	4	0.68	5.87	1.41	0.73	0.70
**60**	U1334B	25.86	14	1.93	7.24	1.41	0.78	2.13
**61**	U1334B	25.86	7	0.73	9.61	1.41	0.84	0.86
**62**	U1334B	25.86	8	1.09	7.35	1.41	0.86	1.32
**63**	U1334B	25.86	17	1.85	9.19	1.41	0.92	2.40
**64**	U1334B	25.86	9	1.17	7.66	1.94	0.94	2.13
**65**	U1334B	25.86	8	0.95	8.38	1.94	0.96	1.77
**66**	U1334B	25.86	9	1.24	7.23	1.94	0.96	2.32
**67**	U1334B	25.86	11	1.34	8.23	1.94	0.95	2.45
**68**	U1334B	25.86	13	1.52	8.54	1.94	0.94	2.77
**69**	U1334B	25.86	7	0.74	9.47	1.94	0.94	1.34
**70**	U1334B	25.86	7	0.84	8.32	1.94	0.95	1.54
**71**	U1334B	25.86	13	1.33	9.77	1.94	0.94	2.43
**72**	U1334B	25.86	8	1.04	7.66	1.94	0.92	1.86
**73**	U1334B	25.86	12	1.30	9.22	1.94	0.93	2.34
**74**	U1334B	25.86	9	1.02	8.8	1.94	0.94	1.87
**75**	U1334B	25.86	26	2.75	9.47	1.94	1.01	5.35
**76**	U1334B	25.86	17	1.95	8.73	1.94	0.95	3.60
**77**	U1334B	25.86	16	1.98	8.09	1.94	0.97	3.70
**78**	U1334B	25.86	10	1.16	8.6	1.94	0.97	2.18
**79**	U1334B	25.86	10	1.11	9.01	1.94	0.95	2.04
**80**	U1334B	25.86	15	1.48	10.13	1.94	1.00	2.87
**81**	U1334B	25.86	30	3.21	9.35	2.01	1.01	6.53
**82**	U1334B	25.86	38	4.02	9.46	2.01	1.01	8.16
**83**	U1334B	25.86	17	2.08	8.16	2.01	0.99	4.14
**84**	U1334B	25.86	13	1.50	8.66	2.01	0.97	2.94
**85**	U1334B	25.86	15	1.66	9.01	2.01	0.96	3.21
**86**	U1334C	25.86	33	1.85	17.88	2.01	0.98	3.64
**87**	U1334C	25.86	33	1.94	17.03	2.01	1.02	3.96
**88**	U1334C	25.86	35	1.91	18.37	2.01	1.05	4.02
**89**	U1334C	25.86	69	3.96	17.44	2.01	1.02	8.15
**90**	U1334C	25.86	35	1.91	18.37	2.01	0.99	3.80
**91**	U1334C	25.86	29	1.69	17.17	2.01	0.99	3.37
**92**	U1334C	25.86	35	2.07	16.88	2.01	0.99	4.11

**93**	U1334C	25.86	62	3.65	16.98	2.01	0.98	7.17
**94**	U1334C	25.86	54	3.00	18.02	2.01	0.98	5.91
**95**	U1334C	25.86	35	2.15	16.27	2.01	0.96	4.16
**96**	U1334C	25.86	52	2.99	17.37	2.01	0.99	5.93
**97**	U1334C	25.86	51	2.90	17.6	2.01	1.00	5.85
**98**	U1334C	25.86	28	1.55	18.04	2.01	0.98	3.07
**99**	U1334C	25.86	22	1.25	17.6	2.01	0.97	2.43
**100**	U1334C	25.86	18	1.01	17.82	2.01	0.95	1.93
**101**	U1334C	25.86	31	1.70	18.28	2.01	1.00	3.40
**102**	U1334C	25.86	21	1.32	15.86	2.01	1.00	2.65
**103**	U1334C	25.86	48	3.65	13.16	2.01	0.94	6.92
**104**	U1334C	25.86	43	2.63	16.35	2.01	0.95	5.01
**105**	U1334C	25.86	39	3.03	12.88	2.01	0.95	5.78
**106**	U1334C	25.86	62	3.53	17.55	2.01	0.96	6.80
**107**	U1334C	25.86	31	2.80	11.07	1.75	0.96	4.69
**108**	U1334C	25.86	44	2.44	18.02	1.75	0.95	4.06
**109**	U1334C	25.86	54	2.62	20.62	1.75	0.97	4.44
**110**	U1334C	25.86	40	2.44	16.41	1.75	0.95	4.07
**111**	U1334C	25.86	24	1.79	13.41	1.75	0.92	2.88
**112**	U1334C	25.86	14	1.00	14.04	1.75	0.92	1.61
**113**	U1334C	25.86	19	1.62	11.74	1.75	0.79	2.23
**114**	U1334C	25.86	10	0.93	10.75	1.75	0.75	1.22
**115**	U1334C	25.86	15	1.32	11.36	1.75	0.73	1.68
**116**	U1334C	25.86	7	0.66	10.59	1.75	0.66	0.76
**117**	U1334C	25.86	12	1.04	11.56	1.75	0.75	1.36
**118**	U1334C	25.86	17	2.05	8.28	1.75	0.77	2.78
**119**	U1334C	25.86	19	1.80	10.58	1.75	0.86	2.71
**120**	U1334C	25.86	27	2.68	10.09	1.75	0.96	4.52
**121**	U1334C	25.86	30	2.78	10.78	1.75	0.93	4.51
**122**	U1334C	25.86	51	5.16	9.88	1.75	0.93	8.38
**123**	U1334C	25.86	29	2.83	10.23	1.75	0.87	4.30
**124**	U1334C	25.86	20	2.38	8.41	1.75	0.83	3.44
**125**	U1334C	25.86	22	1.73	12.71	1.75	0.86	2.62
**126**	U1334C	25.86	12	0.97	12.41	1.75	0.91	1.55
**127**	U1334C	25.86	43	3.37	12.76	1.75	0.97	5.70
**128**	U1334C	25.86	34	3.46	9.82	1.60	0.95	5.29
**129**	U1334C	25.86	24	2.03	11.84	1.60	0.91	2.95
**130**	U1334C	25.86	21	1.96	10.74	1.60	0.95	2.96
**131**	U1334C	25.86	20	1.58	12.67	1.60	0.96	2.42
**132**	U1334C	25.86	32	2.36	13.56	1.60	0.98	3.70
**133**	U1334C	25.86	36	2.93	12.29	1.60	0.98	4.57
**134**	U1334C	25.86	27	2.26	11.96	1.60	0.94	3.39
**135**	U1334C	25.86	18	1.73	10.43	1.60	0.92	2.55
**136**	U1334C	25.86	20	1.68	11.91	1.60	0.96	2.57
**137**	U1334C	25.86	17	1.65	10.3	1.60	0.97	2.57
**138**	U1334C	25.86	14	1.35	10.37	1.60	1.02	2.21
**139**	U1334C	25.86	14	1.49	9.39	1.60	1.03	2.47

**140**	U1334C	25.86	22	1.48	14.82	1.60	1.03	2.44
**141**	U1334C	25.86	31	2.37	13.07	1.60	1.00	3.80
**142**	U1334C	25.86	14	1.06	13.2	1.60	1.05	1.78
**143**	U1334C	25.86	22	1.55	14.21	1.60	1.10	2.73
**144**	U1334C	25.86	19	1.43	13.29	1.60	1.12	2.57
**145**	U1334C	25.86	30	2.20	13.61	1.75	1.10	4.23
**146**	U1334C	25.86	15	1.08	13.84	1.75	1.10	2.09
**147**	U1334C	25.86	37	2.68	13.79	1.75	1.12	5.26
**148**	U1334C	25.86	18	0.79	22.74	1.75	1.13	1.56
**149**	U1334C	25.86	28	2.26	12.41	1.75	1.12	4.39
**150**	U1334C	25.86	29	1.94	14.98	1.75	1.05	3.56
**151**	U1334C	25.86	22	1.73	12.72	1.75	1.07	3.23
**152**	U1334C	25.86	42	2.95	14.22	1.75	1.08	5.58
**153**	U1334C	25.86	34	2.75	12.37	1.75	1.03	4.95
**154**	U1334C	25.86	18	1.40	12.85	1.75	1.02	2.50
**155**	U1334C	25.86	45	3.61	12.46	1.75	0.98	6.17
**156**	U1334C	25.86	23	1.35	17.03	1.75	1.04	2.46
**157**	U1334C	25.86	6	0.51	11.66	1.75	1.00	0.90
**158**	U1334C	25.86	39	3.67	10.64	1.75	0.96	6.14
**159**	U1334B	25.86	30	2.42	12.39	1.75	0.97	4.12
**160**	U1334B	25.86	31	2.40	12.92	1.02	1.01	2.49
**161**	U1334B	25.86	13	1.52	8.54	1.02	0.97	1.50
**162**	U1334B	25.86	14	1.21	11.54	1.02	0.99	1.22
**163**	U1334B	25.86	13	1.27	10.2	1.02	1.00	1.30
**164**	U1334B	25.86	46	2.85	16.16	1.02	0.96	2.79
**165**	U1334B	25.86	24	1.86	12.87	1.02	0.98	1.87
**166**	U1334B	25.86	23	2.02	11.4	1.02	0.98	2.01
**167**	U1334B	25.86	31	2.33	13.29	1.02	0.97	2.31
**168**	U1334B	25.86	28	2.23	12.58	1.02	0.98	2.23
**169**	U1334B	25.86	23	1.89	12.14	1.02	1.00	1.93
**170**	U1334B	25.86	38	2.99	12.71	1.02	1.00	3.07
**171**	U1334B	25.86	30	2.56	11.74	1.02	0.93	2.42
**172**	U1334B	25.86	47	4.04	11.64	1.02	0.96	3.95
**173**	U1334B	25.86	18	1.41	12.74	1.02	0.98	1.41
**174**	U1334B	25.86	29	2.05	14.16	1.02	0.99	2.07
**175**	U1334B	25.86	40	3.75	10.68	1.02	0.94	3.61
**176**	U1334B	25.86	57	4.81	11.85	1.02	0.95	4.69
**177**	U1334B	25.86	46	3.87	11.88	1.02	0.99	3.91
**178**	U1334B	25.86	38	3.36	11.3	1.73	0.96	5.57
**179**	U1334B	25.86	58	4.75	12.2	1.73	0.97	7.99
**180**	U1334B	25.86	38	4.24	8.97	1.73	0.95	6.94
**181**	U1334B	25.86	39	3.96	9.84	1.73	0.95	6.52
**182**	U1334B	25.86	17	2.46	6.92	1.73	0.89	3.78
**183**	U1334B	25.86	34	3.46	9.82	1.73	0.91	5.44
**184**	U1334B	25.86	19	3.01	6.32	1.73	0.93	4.86
**185**	U1334B	25.86	30	3.19	9.41	1.73	0.93	5.13
**186**	U1334B	25.86	17	1.74	9.75	1.73	0.91	2.76

**187**	U1334B	25.86	7	0.81	8.67	1.73	0.95	1.33
**188**	U1334B	25.86	17	2.46	6.92	1.73	0.93	3.97
**189**	U1334B	25.86	14	1.65	8.47	1.73	0.92	2.62
**190**	U1334B	25.86	15	1.85	8.1	1.73	0.87	2.78
**191**	U1334B	25.86	18	1.60	11.25	1.73	0.86	2.37
**192**	U1334B	25.86	7	1.62	4.33	1.73	0.87	2.43
**193**	U1334B	25.86	21	2.10	9.98	1.73	0.89	3.23
**194**	U1334B	25.86	24	2.36	10.18	1.73	0.90	3.69
**195**	U1334B	25.86	30	2.26	13.3	1.73	0.92	3.60
**196**	U1334B	25.86	19	1.90	10.01	1.73	0.90	2.94
**197**	U1334B	25.86	12	1.20	10.02	1.73	0.89	1.85
**198**	U1334B	25.86	13	1.22	10.62	1.73	0.86	1.81
**199**	U1334B	25.86	11	1.30	8.46	1.73	0.87	1.95
**200**	U1334B	25.86	11	1.34	8.23	1.73	0.87	2.02
**201**	U1334B	25.86	16	1.63	9.83	1.73	0.89	2.49
**202**	U1334B	25.86	7	0.50	13.88	1.73	0.88	0.77
**203**	U1334B	25.86	12	1.08	11.14	1.73	0.88	1.63
**204**	U1334B	25.86	12	1.31	9.18	1.73	0.85	1.93
**205**	U1334B	25.86	7	0.60	11.62	1.73	0.86	0.89
**206**	U1334B	25.86	7	0.66	10.68	1.73	0.89	1.01
**207**	U1334B	25.86	8	0.64	12.51	1.73	0.94	1.04
**208**	U1334B	25.86	11	0.90	12.19	1.73	0.94	1.46
**209**	U1334B	25.86	13	1.25	10.38	1.73	0.99	2.14
**210**	U1334B	25.86	16	0.93	17.24	1.73	0.95	1.52
**211**	U1334B	25.86	16	0.93	17.14	1.73	0.88	1.42
**212**	U1334B	25.86	11	0.62	17.63	1.73	0.90	0.98
**213**	U1334B	25.86	28	1.55	18.02	1.15	0.98	1.74
**214**	U1334B	25.86	27	1.47	18.35	1.15	0.95	1.60
**215**	U1334B	25.86	16	0.99	16.15	1.15	0.92	1.04
**216**	U1334B	25.86	30	1.73	17.3	1.15	0.90	1.80
**217**	U1334B	25.86	23	1.30	17.68	1.15	0.89	1.32
**218**	U1334B	25.86	23	1.43	16.08	1.15	0.89	1.47
**219**	U1334B	25.86	36	1.48	24.26	1.15	0.87	1.47
**220**	U1334B	25.86	31	1.29	24.01	1.15	0.89	1.31
**221**	U1334B	25.86	27	1.22	22.11	1.15	0.87	1.22
**222**	U1334B	25.86	27	1.19	22.71	1.15	0.86	1.17
**223**	U1334B	25.86	43	1.87	23.01	1.15	0.83	1.78
**224**	U1334B	25.86	9	0.65	13.92	1.15	0.85	0.63
**225**	U1334B	25.86	24	1.46	16.48	1.15	0.87	1.46
**226**	U1334B	25.86	50	3.32	15.08	1.15	0.89	3.36
**227**	U1334B	25.86	25	1.60	15.66	1.15	0.83	1.51
**228**	U1334B	25.86	14	0.96	14.51	1.15	0.84	0.92
**229**	U1334B	25.86	24	1.51	15.91	1.15	0.77	1.33
**230**	U1334B	25.86	12	0.80	15.01	1.15	0.76	0.69
**231**	U1334B	25.86	21	1.56	13.44	1.15	0.75	1.34
**232**	U1334B	25.86	12	0.68	17.73	1.15	0.70	0.54
**233**	U1334B	25.86	34	1.78	19.15	1.15	0.77	1.57

**234**	U1334B	25.86	37	1.69	21.95	1.15	0.82	1.59
**235**	U1334B	25.86	43	1.74	24.7	1.15	0.89	1.78
**236**	U1334B	25.86	32	1.38	23.22	1.15	0.92	1.45
**237**	U1334C	25.86	32	1.89	16.91	1.54	0.92	2.69
**238**	U1334C	25.86	29	1.77	16.42	1.54	0.92	2.50
**239**	U1334C	25.86	76	2.52	30.15	1.54	0.92	3.57
**240**	U1334C	25.86	77	3.21	24.02	1.54	0.89	4.41
**241**	U1334C	25.86	36	1.49	24.13	1.54	0.89	2.04
**242**	U1334C	25.86	34	1.34	25.41	1.54	0.90	1.86
**243**	U1334C	25.86	16	0.72	22.15	1.54	0.89	0.98
**244**	U1334C	25.86	21	0.75	28.15	1.54	0.86	0.99
**245**	U1334C	25.86	26	1.09	23.91	1.54	0.89	1.49
**246**	U1334C	25.86	34	1.46	23.32	1.54	0.88	1.97
**247**	U1334C	25.86	50	2.32	21.52	1.54	0.84	2.98
**248**	U1334C	25.86	33	1.31	25.16	1.54	0.82	1.66
**249**	U1334C	25.86	49	2.11	23.21	1.54	0.78	2.54
**250**	U1334C	25.86	35	1.76	19.87	1.54	0.83	2.23
**251**	U1334C	25.86	37	1.43	25.93	1.54	0.92	2.01
**252**	U1334C	25.86	40	1.55	25.83	1.54	0.92	2.20
**253**	U1334C	25.86	44	1.68	26.25	1.54	0.88	2.26
**254**	U1334C	25.86	41	1.48	27.67	1.54	0.91	2.08
**255**	U1334C	25.86	49	1.49	32.92	1.54	0.95	2.18
**256**	U1334C	25.86	54	1.84	29.35	1.54	0.97	2.75
**257**	U1334C	25.86	40	1.63	24.59	1.54	0.99	2.48
**258**	U1334C	25.86	51	1.71	29.91	1.54	1.00	2.63
**259**	U1334C	25.86	53	2.39	22.21	1.54	0.97	3.56
**260**	U1334C	25.86	51	2.03	25.16	1.54	1.02	3.18
**261**	U1334C	25.86	40	1.66	24.1	1.54	1.05	2.67
**262**	U1334C	25.86	40	1.52	26.24	1.54	1.06	2.49
**263**	U1334C	25.86	46	1.87	24.66	1.54	1.08	3.09
**264**	U1334C	25.86	54	2.12	25.46	1.54	1.10	3.58
**265**	U1334C	25.86	70	2.61	26.82	1.54	1.14	4.55
**266**	U1334C	25.86	61	1.07	57.1	2.20	1.27	2.98
**267**	U1334C	25.86	27	1.20	22.51	2.20	1.23	3.25
**268**	U1334C	25.86	37	1.54	23.96	2.20	1.33	4.52
**269**	U1334C	25.86	42	1.78	23.6	2.20	1.23	4.83
**270**	U1334C	25.86	31	1.39	22.23	2.20	1.20	3.67
**271**	U1334C	25.86	35	1.56	22.44	2.20	1.11	3.82
**272**	U1334C	25.86	35	1.82	19.23	2.20	1.11	4.43
**273**	U1334C	25.86	48	2.47	19.41	2.20	1.09	5.95
**274**	U1334C	25.86	20	0.90	22.26	2.20	1.12	2.21
**275**	U1334C	25.86	61	2.93	20.81	2.20	1.10	7.10
**276**	U1334C	25.86	34	1.54	22.14	2.20	1.09	3.67
**277**	U1334C	25.86	47	2.07	22.73	2.20	1.17	5.30
**278**	U1334C	25.86	31	1.21	25.55	2.20	1.12	3.00
**279**	U1334C	25.86	34	1.50	22.67	2.20	1.04	3.42
**280**	U1334C	25.86	47	2.00	23.55	2.20	1.01	4.41

**281**	U1334C	25.86	39	1.50	26.02	2.20	0.99	3.26
**282**	U1334C	25.86	33	1.41	23.42	2.20	1.00	3.09
**283**	U1334C	25.86	29	1.45	20.05	2.20	0.97	3.09
**284**	U1334C	25.86	34	1.34	25.4	2.20	0.99	2.92
**285**	U1334C	25.86	40	1.70	23.58	2.20	1.01	3.75
**286**	U1334C	25.86	34	1.13	30.15	2.20	1.00	2.48
**287**	U1334C	25.86	28	1.03	27.14	2.20	0.96	2.18
**288**	U1334C	25.86	25	0.84	29.67	2.20	0.96	1.77
**289**	U1334C	25.86	31	1.08	28.8	2.20	0.95	2.25
**290**	U1334C	25.86	18	0.63	28.68	2.20	0.94	1.29
**291**	U1334C	25.86	17	0.70	24.44	2.20	0.91	1.39
**292**	U1334C	25.86	29	1.10	26.25	2.20	0.87	2.10
**293**	U1334C	25.86	32	1.16	27.67	2.20	0.92	2.33
**294**	U1334C	25.86	14	0.50	27.92	2.20	0.92	1.02
**295**	U1334C	25.86	31	1.28	24.25	2.20	0.92	2.58
**296**	U1334C	25.86	20	1.23	16.29	2.20	0.94	2.53
**297**	U1334C	25.86	16	0.93	17.14	2.20	0.95	1.94
**298**	U1334C	25.86	16	0.96	16.67	1.48	0.95	1.35
**299**	U1334C	25.86	22	1.25	17.57	1.35	0.96	1.62
**300**	U1334C	25.86	25	1.36	18.43	1.35	0.94	1.72
**301**	U1334C	25.86	16	0.89	18.05	1.35	0.91	1.08
**302**	U1334C	25.86	25	1.34	18.6	1.35	0.91	1.65
**303**	U1334C	25.86	23	1.41	16.33	1.35	0.91	1.73
**304**	U1334C	25.86	24	1.43	16.76	1.35	0.89	1.72
**305**	U1334C	25.86	8	0.49	16.31	1.35	0.86	0.57
**306**	U1334C	25.86	15	0.88	16.96	1.35	0.90	1.08
**307**	U1334C	25.86	5	0.30	16.6	1.35	0.90	0.37
**308**	U1334C	25.86	13	0.74	17.64	1.35	0.88	0.87
**309**	U1334C	25.86	18	0.83	21.61	1.35	0.90	1.01
**310**	U1334C	25.86	11	0.44	25.07	1.35	0.93	0.55
**311**	U1334C	25.86	17	0.65	26.21	1.35	0.92	0.81
**312**	U1334C	25.86	11	0.50	21.97	1.35	0.86	0.58
**313**	U1334C	25.86	22	1.21	18.13	1.35	0.90	1.48
**314**	U1334C	25.86	16	0.82	19.44	1.35	0.89	0.99
**315**	U1334C	25.86	32	1.41	22.76	1.35	0.84	1.59
**316**	U1334C	25.86	17	0.80	21.19	1.35	0.86	0.93
**317**	U1334C	25.86	12	0.54	22.3	1.35	0.81	0.59
**318**	U1334C	25.86	13	0.57	22.64	1.35	0.81	0.62
**319**	U1334C	25.86	14	0.74	18.9	1.35	0.79	0.79
**320**	U1334C	25.86	19	1.24	15.3	1.35	0.84	1.40
**321**	U1334C	25.86	20	0.91	21.89	1.35	0.85	1.04
**322**	U1334C	25.86	16	0.87	18.4	1.35	0.84	0.98
**323**	U1334C	25.86	14	0.76	18.54	1.35	0.83	0.84
**324**	U1334C	25.86	25	1.10	22.68	1.35	0.89	1.33
**325**	U1334C	25.86	32	1.33	24.15	1.35	0.91	1.62
**326**	U1334C	25.86	66	2.50	26.41	2.06	0.96	4.95
**327**	U1334C	25.86	665	25.16	26.43	2.06	0.93	48.49

**328**	U1334C	25.86	28	1.16	24.1	2.06	0.88	2.10
**329**	U1334C	25.86	31	1.39	22.23	2.06	0.85	2.44
**330**	U1334C	25.86	22	1.15	19.1	2.06	0.80	1.91
**331**	U1334C	25.86	12	0.65	18.51	2.06	0.79	1.06
**332**	U1334C	25.86	20	1.21	16.48	2.06	0.75	1.88
**333**	U1334C	25.86	8	0.53	15.23	2.06	0.75	0.81
**334**	U1334C	25.86	12	0.81	14.89	2.06	0.86	1.43
**335**	U1334C	25.86	21	0.79	26.43	2.06	0.93	1.53
**336**	U1334C	25.86	18	0.66	27.48	2.06	0.97	1.30
**337**	U1334C	25.86	23	0.89	25.79	2.06	0.97	1.79
**338**	U1334C	25.86	21	0.78	26.96	2.06	0.99	1.58
**339**	U1334C	25.86	36	1.21	29.85	2.06	0.99	2.46
**340**	U1334C	25.86	14	0.55	25.66	2.06	0.97	1.09
**341**	U1334C	25.86	30	1.31	22.91	2.06	0.96	2.60
**342**	U1334C	25.86	24	0.98	24.52	2.06	0.95	1.91
**343**	U1334C	25.86	13	0.57	22.9	2.06	0.93	1.09
**344**	U1334C	25.86	15	0.78	19.15	2.06	0.91	1.47
**345**	U1334C	25.86	23	1.04	22.01	2.06	0.91	1.95
**346**	U1334C	25.86	12	0.58	20.84	2.06	0.90	1.06
**347**	U1334C	25.86	39	1.87	20.86	2.06	0.93	3.58
**348**	U1334C	25.86	19	0.66	28.78	2.06	0.94	1.28
**349**	U1334C	25.86	32	1.17	27.42	2.06	0.90	2.17
**350**	U1334C	25.86	24	0.89	27.05	2.06	0.88	1.60
**351**	U1334C	25.86	41	1.46	28.01	2.06	0.88	2.66
**352**	U1334C	25.86	31	1.06	29.19	2.06	0.93	2.03
**353**	U1334C	25.86	63	2.13	29.64	2.06	0.92	4.04
**354**	U1334C	25.86	48	1.73	27.76	2.06	0.94	3.34
**355**	U1334C	25.86	38	1.39	27.37	2.06	0.94	2.70
**356**	U1334C	25.86	24	1.12	21.45	2.06	0.93	2.15
**357**	U1334C	25.86	16	0.73	21.78	2.06	0.93	1.42
**358**	U1334C	25.86	26	1.17	22.23	2.06	0.92	2.22
**359**	U1334C	25.86	25	0.96	26.11	2.06	0.95	1.87
**360**	U1334C	25.86	34	1.33	25.57	2.06	0.94	2.59
**361**	U1334C	25.86	43	1.56	27.54	2.06	0.95	3.06
**362**	U1334C	25.86	27	1.05	25.82	2.06	0.90	1.94
**363**	U1334C	25.86	30	1.46	20.56	2.06	0.84	2.53
**364**	U1334C	25.86	28	1.25	22.36	2.06	0.89	2.31
**365**	U1334C	25.86	45	1.90	23.72	2.06	0.83	3.24
**366**	U1334C	25.86	16	0.92	17.41	2.06	0.73	1.39
**367**	U1334C	25.86	23	1.00	22.98	2.06	0.76	1.57
**368**	U1334C	25.86	24	1.07	22.39	2.06	0.82	1.81
**369**	U1334C	25.86	37	1.63	22.73	2.06	0.90	3.04
**370**	U1334C	25.86	30	1.20	25.03	2.06	0.97	2.40
**371**	U1334C	25.86	29	1.10	26.45	2.06	0.90	2.03
**372**	U1334C	25.86	25	1.04	24.04	2.06	0.96	2.06
**373**	U1334B	25.86	15	0.81	18.62	1.38	1.00	1.11
**374**	U1334B	25.86	22	1.10	19.95	1.38	1.01	1.53

**375**	U1334B	25.86	29	1.53	18.99	1.38	0.93	1.96
**376**	U1334B	25.86	41	1.71	24.01	1.38	0.92	2.17
**377**	U1334B	25.86	44	1.79	24.52	1.38	0.95	2.36
**378**	U1334B	25.86	24	1.06	22.73	1.38	0.91	1.33
**379**	U1334B	25.86	40	1.71	23.36	1.38	0.88	2.08
**380**	U1334B	25.86	65	2.30	28.26	1.38	0.86	2.73
**381**	U1334B	25.86	41	1.63	25.13	1.38	0.89	1.99
**382**	U1334B	25.86	31	1.21	25.7	1.38	0.97	1.62
**383**	U1334B	25.86	30	1.12	26.75	1.38	0.97	1.49
**384**	U1334B	25.86	19	0.77	24.78	1.38	0.97	1.03
**385**	U1334B	25.86	29	1.20	24.26	1.38	0.98	1.62
**386**	U1334B	25.86	24	1.15	20.95	1.38	1.00	1.57
**387**	U1334B	25.86	24	0.85	28.26	1.38	1.00	1.17
**388**	U1334B	25.86	30	1.19	25.31	1.38	1.04	1.69
**389**	U1334B	25.86	18	0.83	21.62	1.38	1.03	1.18
**390**	U1334B	25.86	23	0.98	23.39	1.38	1.01	1.37
**391**	U1334B	25.86	17	1.15	14.72	1.38	0.98	1.55
**392**	U1334B	25.86	21	1.01	20.74	1.38	1.00	1.40
**393**	U1334B	25.86	26	0.97	26.75	1.38	1.00	1.34
**394**	U1334B	25.86	23	0.86	26.74	1.38	1.01	1.20
**395**	U1334B	25.86	26	1.01	25.85	1.38	1.00	1.38
**396**	U1334B	25.86	26	1.19	21.87	1.38	1.01	1.65
**397**	U1334B	25.86	32	1.20	26.75	1.38	1.01	1.67
**398**	U1334B	25.86	50	2.08	24.05	1.38	1.04	2.97
**399**	U1334B	25.86	29	1.18	24.53	1.38	1.00	1.63
**400**	U1334B	25.86	28	1.53	18.31	1.38	1.00	2.11
**401**	U1334B	25.86	31	1.02	30.3	1.38	1.02	1.44
**402**	U1334B	25.86	27	0.94	28.7	1.38	1.01	1.31
**403**	U1334B	25.86	22	1.05	20.99	1.38	1.02	1.47
**404**	U1334B	25.86	25	0.96	26.1	1.38	1.00	1.32
**405**	U1334B	25.86	12	0.48	25.22	1.38	0.99	0.65
**406**	U1334B	25.86	42	1.49	28.23	1.38	0.99	2.02
**407**	U1334B	25.86	55	2.06	26.73	1.38	0.97	2.76
**408**	U1334B	25.86	39	1.23	31.6	1.38	0.99	1.69
**409**	U1334B	25.86	45	1.71	26.32	1.38	1.00	2.35
**410**	U1334B	25.86	55	2.12	25.99	2.44	0.99	5.10
**411**	U1334B	25.86	37	1.38	26.74	2.44	1.01	3.43
**412**	U1334B	25.86	26	0.94	27.79	2.44	1.00	2.29
**413**	U1334B	25.86	19	0.70	27.22	2.44	0.99	1.69
**414**	U1334B	25.86	26	0.92	28.23	2.44	0.99	2.23
**415**	U1334B	25.86	32	1.43	22.36	2.44	0.97	3.41
**416**	U1334B	25.86	27	1.14	23.75	2.44	0.96	2.67
**417**	U1334B	25.86	38	1.39	27.38	2.44	1.00	3.39
**418**	U1334B	25.86	63	2.66	23.66	2.44	0.99	6.46
**419**	U1334B	25.86	28	1.21	23.16	2.44	0.97	2.86
**420**	U1334B	25.86	27	1.26	21.48	2.44	1.03	3.15
**421**	U1334B	25.86	41	1.97	20.76	2.44	1.00	4.80

**422**	U1334B	25.86	22	1.22	17.98	2.44	0.97	2.90
**423**	U1334B	25.86	22	1.05	20.87	2.44	0.94	2.43
**424**	U1334B	25.86	19	0.88	21.62	2.44	0.91	1.96
**425**	U1334B	25.86	20	0.94	21.24	2.44	0.91	2.08
**426**	U1334B	25.86	14	0.69	20.31	2.44	0.91	1.53
**427**	U1334B	25.86	40	2.22	18.02	2.44	0.93	5.04
**428**	U1334B	25.86	6	0.28	21.15	2.44	0.93	0.65
**429**	U1334B	25.86	8	0.36	22.27	2.44	0.97	0.85
**430**	U1334B	25.86	23	1.02	22.57	2.44	0.98	2.43
**431**	U1334B	25.86	11	0.50	21.97	2.44	1.00	1.22
**432**	U1334B	25.86	12	0.57	21.07	2.44	0.99	1.38
**433**	U1334B	25.86	17	0.74	23	2.44	0.96	1.74
**434**	U1334B	25.86	19	0.89	21.33	2.44	0.97	2.11
**435**	U1334B	25.86	12	0.57	21.05	2.44	0.95	1.32
**436**	U1334B	25.86	12	0.51	23.76	2.44	0.97	1.20
**437**	U1334B	25.86	20	0.90	22.15	2.44	0.96	2.11
**438**	U1334B	25.86	12	0.48	24.86	2.44	0.97	1.14
**439**	U1334B	25.86	20	0.75	26.7	2.44	1.01	1.84
**440**	U1334B	25.86	7	0.27	25.55	2.44	1.03	0.69
**441**	U1334B	25.86	10	0.36	28.06	2.44	1.02	0.89
**442**	U1334B	25.86	12	0.42	28.68	2.44	1.04	1.06
**443**	U1334B	25.86	8	0.34	23.44	2.44	1.04	0.87
**444**	U1334B	25.86	20	0.86	23.28	2.44	1.07	2.25
**445**	U1334B	25.86	12	0.53	22.65	2.44	1.08	1.40
**446**	U1334B	25.86	20	0.81	24.65	2.44	1.07	2.11
**447**	U1334B	25.86	8	0.35	23.12	2.44	1.09	0.92
**448**	U1334B	25.86	36	1.61	22.42	2.44	1.08	4.23
**449**	U1334B	25.86	30	1.06	28.33	2.44	1.05	2.71
**450**	U1334B	25.86	11	0.50	22.15	2.44	1.05	1.28
**451**	U1334B	25.86	21	0.89	23.63	2.44	1.07	2.32
**452**	U1334B	25.86	14	0.69	20.25	2.44	1.05	1.77
**453**	U1334B	25.86	13	0.64	20.4	2.44	1.06	1.65
**454**	U1334B	25.86	8	0.52	15.26	1.60	1.08	0.90
**455**	U1334B	25.86	10	0.44	22.92	1.60	1.10	0.76
**456**	U1334B	25.86	8	0.34	23.39	1.60	1.08	0.59
**457**	U1334B	25.86	18	0.74	24.45	1.60	1.00	1.17
**458**	U1334B	25.86	23	0.94	24.51	1.60	1.06	1.59
**459**	U1334B	25.86	9	0.35	25.8	1.60	1.06	0.59
**460**	U1334B	25.86	26	0.95	27.25	1.60	1.07	1.63
**461**	U1334B	25.86	11	0.43	25.73	1.60	1.04	0.71
**462**	U1334B	25.86	9	0.35	25.64	1.60	1.04	0.58
**463**	U1334B	25.86	13	0.50	26.07	1.60	1.04	0.83
**464**	U1334B	25.86	36	1.35	26.69	1.60	1.02	2.19
**465**	U1334B	25.86	13	0.55	23.82	1.60	1.02	0.89
**466**	U1334B	25.86	19	0.78	24.3	1.60	1.03	1.28
**467**	U1334B	25.86	18	0.79	22.79	1.60	1.00	1.27
**468**	U1334B	25.86	7	0.30	23.42	1.60	1.01	0.48

**469**	U1334B	25.86	8	0.30	26.63	1.60	1.04	0.50
**470**	U1334C	25.86	11	0.40	27.68	1.60	1.00	0.63
**471**	U1334C	25.86	10	0.31	32.57	1.60	1.05	0.51
**472**	U1334C	25.86	28	0.89	31.42	1.60	1.06	1.50
**473**	U1334C	25.86	8	0.27	29.68	1.60	1.05	0.45
**474**	U1334C	25.86	18	0.54	33.04	1.60	1.03	0.90
**475**	U1334C	25.86	25	0.63	39.54	1.60	1.02	1.03
**476**	U1334C	25.86	36	1.00	36.17	1.60	0.98	1.57
**477**	U1334C	25.86	30	1.01	29.75	1.60	0.95	1.53
**478**	U1334C	25.86	28	1.00	27.99	1.60	0.93	1.49
**479**	U1334C	25.86	21	0.52	40.42	1.60	0.99	0.82
**480**	U1334C	25.86	28	0.94	29.9	1.60	0.96	1.43
**481**	U1334C	25.86	40	1.33	30	1.60	0.96	2.05
**482**	U1334C	25.86	36	1.28	28.03	1.60	0.96	1.97
**483**	U1334C	25.86	24	0.86	27.93	1.60	0.91	1.25
**484**	U1334C	25.86	24	0.98	24.57	1.60	1.01	1.58
**485**	U1334C	25.86	42	1.60	26.23	1.60	1.00	2.55
**486**	U1334C	25.86	24	1.08	22.13	1.60	0.99	1.71
**487**	U1334C	25.86	27	1.12	24.09	1.60	0.96	1.72
**488**	U1334C	25.86	12	0.61	19.55	1.60	0.97	0.96
**489**	U1334C	25.86	21	0.91	22.96	1.60	0.99	1.45
**490**	U1334C	25.86	36	1.75	20.56	2.10	0.99	3.65
**491**	U1334C	25.86	18	0.98	18.4	2.10	1.01	2.06
**492**	U1334C	25.86	43	1.46	29.42	2.10	1.02	3.11
**493**	U1334C	25.86	33	1.31	25.26	2.10	1.01	2.76
**494**	U1334C	25.86	38	1.21	31.44	2.10	0.97	2.45
**495**	U1334C	25.86	25	0.90	27.81	2.10	0.96	1.82
**496**	U1334C	25.86	38	1.34	28.27	2.10	0.93	2.62
**497**	U1334C	25.86	29	1.02	28.52	2.10	0.94	2.00
**498**	U1334C	25.86	16	0.60	26.7	2.10	0.94	1.18
**499**	U1334C	25.86	19	0.92	20.74	2.10	0.91	1.75
**500**	U1334C	25.86	18	0.86	20.91	2.10	0.95	1.71
**501**	U1334C	25.86	16	1.00	16.07	2.10	0.94	1.95
**502**	U1334C	25.86	17	1.17	14.54	2.10	0.89	2.19
**503**	U1334C	25.86	17	0.74	23	2.10	0.89	1.38
**504**	U1334C	25.86	11	0.48	22.73	2.10	0.89	0.90
**505**	U1334C	25.86	8	0.28	28.7	2.10	0.88	0.51
**506**	U1334C	25.86	29	1.31	22.16	2.10	0.89	2.43
**507**	U1334C	25.86	8	0.32	24.88	2.10	0.89	0.60
**508**	U1334C	25.86	12	0.46	26.01	2.10	0.91	0.88
**509**	U1334C	25.86	16	0.52	30.57	2.10	0.91	1.00
**510**	U1334C	25.86	30	1.17	25.54	2.10	0.90	2.22
**511**	U1334C	25.86	14	0.56	25.15	2.10	0.91	1.06
**512**	U1334C	25.86	8	0.30	26.81	2.10	0.89	0.56
**513**	U1334C	25.86	16	0.76	20.94	2.10	0.95	1.52
**514**	U1334C	25.86	17	0.69	24.73	2.10	0.94	1.36
**515**	U1334C	25.86	19	0.88	21.52	2.10	0.96	1.78

**516**	U1334C	25.86	13	0.61	21.32	2.10	0.92	1.18
**517**	U1334C	25.86	36	1.73	20.82	2.10	0.90	3.27
**518**	U1334C	25.86	16	0.74	21.73	2.10	0.93	1.43
**519**	U1334C	25.86	15	0.65	22.99	2.10	0.91	1.24
**520**	U1334C	25.86	14	0.71	19.59	2.10	0.90	1.35
**521**	U1334C	25.86	11	0.52	21.02	2.10	0.87	0.96
**522**	U1334C	25.86	10	0.39	25.58	2.10	0.90	0.73
**523**	U1334C	25.86	20	0.81	24.55	2.10	0.95	1.62
**524**	U1334C	25.86	13	0.46	28.28	2.10	0.94	0.91
**525**	U1334C	25.86	7	0.30	23.6	2.10	0.94	0.59
**526**	U1334C	25.86	7	0.32	22.16	2.10	0.99	0.66
**527**	U1334C	25.86	15	0.50	30	2.10	0.97	1.01
**528**	U1334C	25.86	15	0.48	31.5	2.10	0.98	0.98
**529**	U1334C	25.86	19	0.73	25.99	2.10	0.99	1.51
**530**	U1334C	25.86	28	1.02	27.45	2.10	1.01	2.16
**531**	U1334C	25.86	9	0.35	25.88	2.10	1.01	0.74
**532**	U1334C	25.86	28	1.13	24.88	2.35	1.03	2.71
**533**	U1334C	25.86	16	0.68	23.44	2.35	1.02	1.64
**534**	U1334C	25.86	16	0.59	26.9	2.35	1.00	1.40
**535**	U1334C	25.86	13	0.55	23.7	2.35	0.99	1.27
**536**	U1334C	25.86	23	0.91	25.17	2.35	0.95	2.05
**537**	U1334C	25.86	16	0.71	22.67	2.35	0.90	1.49
**538**	U1334C	25.86	21	0.56	37.26	2.35	0.89	1.18
**539**	U1334C	25.86	14	0.52	26.81	2.35	0.94	1.16
**540**	U1334C	25.86	26	1.02	25.5	2.35	0.98	2.35
**541**	U1334C	25.86	18	0.90	20.11	2.35	1.01	2.13
**542**	U1334C	25.86	9	0.55	16.29	2.35	1.01	1.31
**543**	U1334C	25.86	15	0.62	24.1	2.35	1.04	1.52
**544**	U1334C	25.86	13	0.50	25.9	2.35	1.03	1.22
**545**	U1334C	25.86	25	1.01	24.64	2.35	1.04	2.48
**546**	U1334C	25.86	35	1.49	23.47	2.35	1.05	3.68
**547**	U1334C	25.86	36	1.60	22.45	2.35	1.02	3.85
**548**	U1334C	25.86	47	2.38	19.74	2.35	1.02	5.69
**549**	U1334C	25.86	13	0.53	24.55	2.35	0.98	1.21
**550**	U1334C	25.86	10	0.48	20.98	2.35	0.94	1.05
**551**	U1334C	25.86	10	0.37	26.83	2.35	0.94	0.82
**552**	U1334C	25.86	17	0.66	25.66	2.35	0.96	1.50
**553**	U1334C	25.86	17	0.71	24.05	2.35	0.98	1.63
**554**	U1334C	25.86	15	0.56	26.92	2.35	0.98	1.29
**555**	U1334C	25.86	17	0.57	29.58	2.35	0.94	1.28
**556**	U1334C	25.86	27	0.82	32.87	2.35	0.98	1.89
**557**	U1334C	25.86	11	0.37	29.63	2.35	0.99	0.87
**558**	U1334C	25.86	27	0.85	31.86	2.35	0.95	1.90
**559**	U1334C	25.86	14	0.47	30.05	2.35	0.96	1.05
**560**	U1334C	25.86	22	0.73	30.07	2.35	0.96	1.66
**561**	U1334C	25.86	29	1.01	28.58	2.35	0.94	2.23
**562**	U1334C	25.86	52	1.68	30.94	2.35	0.92	3.66

**563**	U1334C	25.86	26	0.92	28.29	2.35	0.92	1.98
**564**	U1334C	25.86	23	0.79	29.13	2.35	0.93	1.72
**565**	U1334C	25.86	29	0.99	29.19	2.35	0.97	2.27
**566**	U1334C	25.86	118	3.20	36.91	2.35	0.96	7.25
**567**	U1334C	25.86	19	0.59	32.11	2.35	0.94	1.31
**568**	U1334C	25.86	66	2.40	27.53	2.35	0.98	5.54
**569**	U1334C	25.86	41	1.44	28.4	2.35	1.02	3.48
**570**	U1334C	25.86	47	1.67	28.13	2.35	1.01	3.97
**571**	U1334C	25.86	32	1.27	25.22	1.75	1.02	2.25
**572**	U1334C	25.86	22	1.06	20.76	1.04	1.04	1.15
**573**	U1334C	25.86	19	0.95	19.97	1.04	1.02	1.02
**574**	U1334C	25.86	24	1.00	24.12	1.04	1.01	1.05
**575**	U1334C	25.86	24	0.85	28.27	1.04	1.01	0.89
**576**	U1334C	25.86	35	1.12	31.13	1.04	0.98	1.15
**577**	U1334C	25.86	13	0.46	28.47	1.04	0.98	0.47
**578**	U1334C	25.86	15	0.67	22.23	1.04	1.00	0.71
**579**	U1334C	25.86	21	0.87	24.07	1.04	1.01	0.92
**580**	U1334C	25.86	20	0.89	22.42	1.04	1.02	0.95
**581**	U1334C	25.86	18	0.67	26.97	1.04	1.02	0.71
**582**	U1334C	25.86	32	1.13	28.2	1.04	1.00	1.18
**583**	U1334C	25.86	24	1.08	22.29	1.04	0.99	1.12
**584**	U1334C	25.86	42	1.46	28.77	1.04	1.03	1.56
**585**	U1334C	25.86	38	1.47	25.84	1.04	0.95	1.46
**586**	U1334C	25.86	62	2.38	26.04	1.04	0.98	2.43
**587**	U1334C	25.86	39	1.33	29.28	1.04	0.98	1.36
**588**	U1334C	25.86	25	0.86	29.15	1.04	1.00	0.90
**589**	U1334C	25.86	28	1.10	25.44	1.04	0.98	1.13
**590**	U1334B	25.86	39	1.57	24.83	1.04	1.01	1.65
**591**	U1334B	25.86	23	0.81	28.28	0.82	1.03	0.68
**592**	U1334B	25.86	20	0.82	24.36	0.82	1.02	0.68
**593**	U1334B	25.86	25	0.93	26.86	0.82	0.97	0.74
**594**	U1334B	25.86	13	0.49	26.76	0.82	0.96	0.38
**595**	U1334B	25.86	10	0.41	24.68	0.82	0.96	0.32
**596**	U1334B	25.86	13	0.58	22.31	0.82	0.96	0.46
**597**	U1334B	25.86	16	0.67	23.73	0.82	0.98	0.54
**598**	U1334B	25.86	10	0.42	23.67	0.82	0.95	0.33
**599**	U1334B	25.86	11	0.40	27.62	0.82	0.93	0.30
**600**	U1334B	25.86	18	0.81	22.29	0.82	0.94	0.62
**601**	U1334B	25.86	15	0.63	23.63	0.82	0.93	0.48
**602**	U1334B	25.86	23	0.96	23.99	0.82	0.90	0.70
**603**	U1334B	25.86	36	1.55	23.16	0.82	0.96	1.22
**604**	U1334B	25.86	17	0.70	24.15	0.82	0.94	0.54
**605**	U1334B	25.86	26	1.02	25.46	0.82	0.95	0.79
**606**	U1334B	25.86	23	0.79	29.17	0.82	0.98	0.63
**607**	U1334B	25.86	25	0.90	27.8	0.82	0.99	0.73
**608**	U1334B	25.86	24	0.88	27.29	1.86	1.00	1.63
**609**	U1334B	25.86	28	1.05	26.78	1.86	1.03	1.99

**610**	U1334B	25.86	68	2.34	29.07	1.86	1.01	4.40
**611**	U1334B	25.86	20	0.84	23.91	1.86	1.01	1.57
**612**	U1334B	25.86	20	0.76	26.22	1.86	1.01	1.43
**613**	U1334B	25.86	17	0.57	29.86	1.86	1.02	1.07
**614**	U1334B	25.86	11	0.41	27.13	1.86	1.02	0.77
**615**	U1334B	25.86	29	1.08	26.73	1.86	1.00	2.01
**616**	U1334B	25.86	13	0.57	22.86	1.86	1.00	1.05
**617**	U1334B	25.86	14	0.48	29.22	1.86	1.03	0.92
**618**	U1334B	25.86	16	0.68	23.48	1.86	1.02	1.29
**619**	U1334B	25.86	26	0.93	28	1.86	0.99	1.71
**620**	U1334B	25.86	12	0.51	23.57	1.86	0.99	0.94
**621**	U1334B	25.86	23	0.89	25.92	1.86	0.98	1.62
**622**	U1334B	25.86	25	0.96	26.1	1.86	0.98	1.74
**623**	U1334B	25.86	23	0.77	29.74	1.86	1.01	1.46
**624**	U1334B	25.86	18	0.80	22.64	1.86	0.99	1.46
**625**	U1334B	25.86	34	1.53	22.16	1.86	0.99	2.83
**626**	U1334B	25.86	10	0.47	21.35	1.86	0.93	0.81
**627**	U1334B	25.86	17	0.90	18.95	1.86	0.91	1.52
**628**	U1334B	25.86	20	0.96	20.73	1.86	0.91	1.62
**629**	U1334B	25.86	16	0.68	23.37	1.86	0.98	1.25
**630**	U1334B	25.86	17	0.67	25.48	1.86	1.03	1.27
**631**	U1334B	25.86	12	0.52	23.03	1.86	1.01	0.98
**632**	U1334B	25.86	13	0.50	26.21	1.86	1.03	0.95
**633**	U1334B	25.86	20	0.79	25.35	1.86	1.02	1.50
**634**	U1334B	25.86	29	1.16	24.98	1.86	1.01	2.19
**635**	U1334B	25.86	32	1.24	25.8	1.86	1.01	2.32
**636**	U1334B	25.86	27	1.18	22.91	1.86	1.06	2.32
**637**	U1334B	25.86	31	1.29	24.06	1.86	0.99	2.37
**638**	U1334B	25.86	29	1.28	22.59	1.86	1.03	2.45
**639**	U1334B	25.86	76	3.09	24.63	1.86	1.02	5.87
**640**	U1334B	25.86	31	1.28	24.28	1.86	1.05	2.50
**641**	U1334B	25.86	18	0.98	18.42	1.86	1.01	1.83
**642**	U1334B	25.86	15	0.56	26.6	1.86	1.03	1.08
**643**	U1334B	25.86	29	1.31	22.22	1.86	1.05	2.55
**644**	U1334B	25.86	21	1.06	19.76	1.86	1.05	2.07
**645**	U1334B	25.86	13	0.56	23.41	1.86	1.05	1.09
**646**	U1334B	25.86	18	0.97	18.63	1.86	1.12	2.01
**647**	U1334B	25.86	14	0.61	23.06	2.53	1.08	1.66
**648**	U1334B	25.86	42	1.94	21.69	2.53	1.11	5.44
**649**	U1334B	25.86	39	1.60	24.4	2.53	1.07	4.34
**650**	U1334B	25.86	21	1.02	20.6	2.53	1.08	2.78
**651**	U1334B	25.86	18	0.80	22.53	2.53	1.10	2.23
**652**	U1334B	25.86	15	0.69	21.71	2.53	1.10	1.91
**653**	U1334B	25.86	16	0.64	24.93	2.53	1.11	1.80
**654**	U1334B	25.86	27	1.03	26.14	2.53	1.10	2.88
**655**	U1334B	25.86	18	0.76	23.55	2.53	1.06	2.05
**656**	U1334B	25.86	25	0.99	25.14	2.53	1.07	2.69

**657**	U1334B	25.86	20	0.84	23.87	2.53	1.08	2.28
**658**	U1334B	25.86	19	0.79	24.11	2.53	1.08	2.15
**659**	U1334B	25.86	32	1.31	24.44	2.53	1.03	3.39
**660**	U1334B	25.86	28	1.25	22.48	2.53	0.99	3.13
**661**	U1334B	25.86	33	1.38	23.87	2.53	1.02	3.57
**662**	U1334B	25.86	12	0.55	21.99	2.53	1.00	1.37
**663**	U1334B	25.86	9	0.40	22.3	2.53	1.01	1.03
**664**	U1334B	25.86	15	0.65	22.95	2.53	0.98	1.62
**665**	U1334B	25.86	25	0.92	27.05	2.53	1.02	2.39
**666**	U1334B	25.86	20	0.74	26.93	2.53	1.04	1.95
**667**	U1334B	25.86	15	0.60	25.04	2.53	1.05	1.59
**668**	U1334B	25.86	13	0.47	27.5	2.53	1.06	1.26
**669**	U1334B	25.86	24	0.86	27.91	2.53	1.01	2.20
**670**	U1334B	25.86	12	0.55	21.85	2.53	1.00	1.39
**671**	U1334B	25.86	11	0.44	25.15	2.53	0.97	1.07
**672**	U1334B	25.86	11	0.54	20.28	2.53	0.96	1.32
**673**	U1334B	25.86	27	1.39	19.46	2.53	0.94	3.31
**674**	U1334B	25.86	17	0.69	24.64	2.53	0.99	1.72
**675**	U1334B	25.86	32	1.34	23.96	2.53	0.99	3.34
**676**	U1334B	25.86	32	1.24	25.77	2.53	0.99	3.10
**677**	U1334B	25.86	36	1.41	25.58	2.53	1.03	3.67
**678**	U1334B	25.86	39	1.50	26	2.53	1.09	4.13
**679**	U1334B	25.86	15	0.57	26.17	2.53	1.10	1.59
**680**	U1334B	25.86	22	0.87	25.34	2.53	1.13	2.47
**681**	U1334B	25.86	39	1.62	24.06	2.53	1.12	4.58
**682**	U1334B	25.86	35	1.42	24.73	2.53	1.07	3.84
**683**	U1334B	25.86	20	0.97	20.58	2.53	1.02	2.52
**684**	U1334B	25.86	36	1.55	23.28	2.53	1.06	4.13
**685**	U1334B	25.86	26	1.45	17.94	2.53	1.05	3.85
**686**	U1334B	25.86	24	1.12	21.36	2.53	1.06	3.02
**687**	U1334B	25.86	12	0.61	19.53	2.53	1.06	1.64
**688**	U1334B	25.86	11	0.52	21.22	2.53	1.07	1.40
**689**	U1334B	25.86	19	0.95	19.9	2.53	1.07	2.58
**690**	U1334B	25.86	23	0.95	24.28	2.53	1.10	2.62
**691**	U1334B	25.86	17	0.59	29.05	2.53	1.09	1.62
**692**	U1334B	25.86	16	0.60	26.54	2.53	1.07	1.62
**693**	U1334B	25.86	19	0.71	26.68	2.53	1.07	1.92
**694**	U1334B	25.86	36	1.28	28.22	2.53	1.07	3.45
**695**	U1334B	25.86	54	2.00	26.94	2.53	1.11	5.61
**696**	U1334B	25.86	15	0.56	26.88	2.53	1.10	1.55
**697**	U1334B	25.86	36	1.42	25.37	2.53	1.08	3.86
**698**	U1334B	25.86	26	1.09	23.82	2.53	1.10	3.04
**699**	U1334B	25.86	24	0.99	24.31	2.53	1.10	2.75
**700**	U1334B	25.86	25	1.00	24.89	2.53	1.06	2.70
**701**	U1334B	25.86	21	0.82	25.68	2.53	1.08	2.24
**702**	U1334B	25.86	30	1.11	27.04	2.53	1.09	3.06
**703**	U1334B	25.86	28	1.18	23.74	2.53	1.13	3.37

**704**	U1334B	25.86	14	0.51	27.47	2.53	1.12	1.44
**705**	U1334B	25.86	25	0.93	26.8	2.53	1.12	2.65
**706**	U1334B	25.86	24	0.85	28.14	2.53	1.11	2.39
**707**	U1334B	25.86	21	0.78	26.81	2.53	1.09	2.15
**708**	U1334B	25.86	24	1.16	20.77	2.53	1.09	3.17
**709**	U1334B	25.86	23	1.13	20.36	2.53	1.09	3.12
**710**	U1334B	25.86	17	0.75	22.7	2.53	1.09	2.06
**711**	U1334B	25.86	28	1.23	22.77	2.53	1.08	3.36
**712**	U1334B	25.86	19	0.74	25.84	2.53	1.12	2.08
**713**	U1334B	25.86	36	1.41	25.58	2.53	1.13	4.01
**714**	U1334B	25.86	35	1.28	27.25	2.53	1.13	3.65
**715**	U1334B	25.86	34	1.31	26.04	2.53	1.14	3.75
**716**	U1334B	25.86	93	4.37	21.28	2.53	1.13	12.48
**717**	U1334B	25.86	23	1.00	23.06	2.53	1.10	2.78
**718**	U1334B	25.86	33	1.45	22.73	2.53	1.12	4.10
**719**	U1334B	25.86	23	1.03	22.23	2.53	1.11	2.91
**720**	U1334B	25.86	23	0.94	24.57	2.53	1.12	2.65
**721**	U1334B	25.86	21	0.98	21.4	2.53	1.11	2.76
**722**	U1334B	25.86	59	2.28	25.88	2.53	1.13	6.50
**723**	U1334B	25.86	32	1.25	25.55	2.40	1.13	3.40
**724**	U1334B	25.86	48	1.71	27.99	2.38	1.10	4.48
**725**	U1334B	25.86	40	1.55	25.82	2.38	1.11	4.09
**726**	U1334B	25.86	25	0.90	27.7	2.38	1.13	2.41
**727**	U1334B	25.86	26	0.82	31.54	2.38	1.10	2.16
**728**	U1334B	25.86	38	1.05	36.11	2.38	1.10	2.75
**729**	U1334B	25.86	27	0.91	29.63	2.38	1.09	2.37
**730**	U1334B	25.86	25	0.83	30.24	2.38	1.07	2.10
**731**	U1334B	25.86	26	0.67	38.79	2.38	1.05	1.68
**732**	U1334B	25.86	25	0.91	27.61	2.38	1.03	2.22
**733**	U1334B	25.86	18	0.63	28.43	2.38	1.03	1.55
**734**	U1334B	25.86	20	0.72	27.69	2.38	1.00	1.72
**735**	U1334B	25.86	29	1.07	27.18	2.38	1.03	2.60
**736**	U1334B	25.86	21	0.83	25.3	2.38	1.01	2.00
**737**	U1334C	25.86	17	0.63	26.99	2.38	1.02	1.53
**738**	U1334C	25.86	25	1.20	20.79	2.38	0.94	2.69
**739**	U1334C	25.86	12	0.50	23.92	2.38	0.94	1.12
**740**	U1334C	25.86	19	0.79	24	2.38	0.97	1.82
**741**	U1334C	25.86	13	0.52	25.21	2.38	0.93	1.15
**742**	U1334C	25.86	17	0.63	27.12	2.38	0.97	1.45
**743**	U1334C	25.86	23	0.84	27.32	2.38	0.98	1.97
**744**	U1334C	25.86	27	1.05	25.75	2.38	1.01	2.53
**745**	U1334C	25.86	33	1.19	27.65	2.38	1.00	2.85
**746**	U1334C	25.86	29	1.10	26.29	2.38	1.02	2.66
**747**	U1334C	25.86	20	0.67	29.82	2.38	0.99	1.58
**748**	U1334C	25.86	20	0.80	25.02	2.38	0.94	1.79
**749**	U1334C	25.86	16	0.61	26.35	2.38	0.91	1.32
**750**	U1334C	25.86	23	0.84	27.48	2.38	0.95	1.89

**751**	U1334C	25.86	14	0.65	21.41	2.38	0.94	1.45
**752**	U1334C	25.86	16	0.70	22.85	2.38	0.94	1.57
**753**	U1334C	25.86	22	0.91	24.25	2.38	1.01	2.18
**754**	U1334C	25.86	22	0.96	22.91	2.38	1.02	2.32
**755**	U1334C	25.86	12	0.57	21.1	2.38	1.00	1.36
**756**	U1334C	25.86	15	0.67	22.41	2.38	1.02	1.63
**757**	U1334C	25.86	28	1.20	23.34	2.38	1.02	2.91
**758**	U1334C	25.86	23	1.11	20.68	2.38	1.03	2.73
**759**	U1334C	25.86	30	1.31	22.85	2.38	1.04	3.23
**760**	U1334C	25.86	17	0.70	24.29	2.38	1.07	1.78
**761**	U1334C	25.86	19	0.81	23.49	2.38	1.03	1.97
**762**	U1334C	25.86	17	0.76	22.35	2.38	1.04	1.88
**763**	U1334C	25.86	18	0.71	25.35	2.38	1.07	1.81
**764**	U1334C	25.86	20	0.86	23.31	2.38	1.08	2.21
**765**	U1334C	25.86	28	1.35	20.69	2.38	1.04	3.34
**766**	U1334C	25.86	33	1.52	21.76	2.38	1.03	3.70
**767**	U1334C	25.86	13	0.71	18.3	2.38	1.03	1.74
**768**	U1334C	25.86	16	0.76	20.93	2.38	1.06	1.92
**769**	U1334C	25.86	24	0.93	25.77	2.38	1.07	2.37
**770**	U1334C	25.86	20	0.86	23.26	2.38	1.11	2.26
**771**	U1334C	25.86	20	0.80	25.03	2.38	1.09	2.07
**772**	U1334C	25.86	12	0.49	24.74	2.38	1.08	1.25
**773**	U1334C	25.86	16	0.73	21.81	2.38	1.12	1.95
**774**	U1334C	25.86	17	0.65	26.23	2.38	1.09	1.68
**775**	U1334C	25.86	22	0.85	25.77	2.38	1.12	2.28
**776**	U1334C	25.86	30	1.24	24.19	2.37	1.11	3.25
**777**	U1334C	25.86	19	0.71	26.75	2.37	1.10	1.85
**778**	U1334C	25.86	29	1.24	23.41	2.37	1.07	3.15
**779**	U1334C	25.86	14	0.73	19.25	2.37	1.10	1.89
**780**	U1334C	25.86	18	0.67	27.05	2.37	1.08	1.70
**781**	U1334C	25.86	26	1.04	25.1	2.37	1.08	2.66
**782**	U1334C	25.86	50	2.26	22.08	2.37	1.07	5.74
**783**	U1334C	25.86	29	1.11	26.03	2.37	1.06	2.81
**784**	U1334C	25.86	29	1.26	23.03	2.37	1.09	3.25
**785**	U1334C	25.86	46	1.78	25.91	2.37	1.04	4.36
**786**	U1334C	25.86	25	1.29	19.45	2.37	1.02	3.10
**787**	U1334C	25.86	37	1.83	20.23	2.37	1.03	4.44
**788**	U1334C	25.86	34	1.68	20.22	2.37	1.01	4.00
**789**	U1334C	25.86	30	1.44	20.82	2.37	1.03	3.51
**790**	U1334C	25.86	14	0.59	23.8	2.37	1.03	1.43
**791**	U1334C	25.86	22	0.88	24.92	2.37	1.04	2.17
**792**	U1334C	25.86	18	0.80	22.48	2.37	1.07	2.03
**793**	U1334C	25.86	24	1.13	21.21	2.37	1.14	3.05
**794**	U1334C	25.86	32	1.63	19.6	2.37	1.15	4.43
**795**	U1334C	25.86	30	1.50	20.04	2.37	1.10	3.92
**796**	U1334C	25.86	18	1.08	16.66	2.37	1.09	2.79
**797**	U1334C	25.86	20	0.82	24.48	2.37	1.04	2.01

**798**	U1334C	25.86	20	0.95	21.13	2.37	1.05	2.36
**799**	U1334C	25.86	11	0.56	19.78	2.37	1.03	1.36
**800**	U1334C	25.86	23	1.12	20.56	2.37	1.00	2.65
**801**	U1334C	25.86	21	0.88	23.82	2.37	1.00	2.08
**802**	U1334C	25.86	25	0.62	40.04	2.37	0.98	1.44
**803**	U1334C	25.86	14	0.58	24.28	2.37	0.98	1.34
**804**	U1334C	25.86	24	1.05	22.84	2.37	0.98	2.43
**805**	U1334C	25.86	24	1.01	23.72	2.37	1.04	2.49
**806**	U1334C	25.86	24	1.06	22.55	2.37	1.03	2.58
**807**	U1334C	25.86	13	0.54	24.13	2.37	1.08	1.37
**808**	U1334B	25.86	40	1.50	26.65	2.37	1.10	3.90
**809**	U1334B	25.86	39	1.40	27.76	2.37	1.12	3.71
**810**	U1334B	25.86	16	0.59	26.99	2.37	1.09	1.53
**811**	U1334B	25.86	33	1.22	26.95	2.37	1.11	3.21
**812**	U1334B	25.86	21	0.87	24.11	2.37	1.08	2.22
**813**	U1334B	25.86	32	1.26	25.35	2.37	1.10	3.30
**814**	U1334B	25.86	48	1.79	26.75	2.37	1.09	4.63
**815**	U1334B	25.86	29	0.81	36.02	2.37	1.08	2.05
**816**	U1334B	25.86	22	0.95	23.15	2.37	1.10	2.48
**817**	U1334B	25.86	14	0.54	26	2.37	1.11	1.41
**818**	U1334B	25.86	24	0.94	25.54	2.37	1.12	2.50
**819**	U1334B	25.86	39	1.26	30.85	2.37	1.12	3.36
**820**	U1334B	25.86	41	1.53	26.72	2.37	1.08	3.92
**821**	U1334B	25.86	22	0.87	25.25	2.37	1.09	2.24
**822**	U1334B	25.86	23	0.79	28.94	2.37	1.08	2.02
**823**	U1334B	25.86	22	0.68	32.51	2.37	1.07	1.71
**824**	U1334B	25.86	13	0.51	25.52	2.37	1.07	1.29
**825**	U1334B	25.86	115	3.38	34.02	2.37	1.10	8.80
**826**	U1334B	25.86	36	1.18	30.59	1.85	1.10	2.40
**827**	U1334B	25.86	14	0.42	33.03	1.64	1.07	0.74
**828**	U1334B	25.86	15	0.48	31.56	1.64	1.05	0.82
**829**	U1334B	25.86	48	1.73	27.75	1.64	1.08	3.07
**830**	U1334B	25.86	22	0.77	28.49	1.64	1.01	1.29
**831**	U1334B	25.86	40	1.26	31.85	1.64	1.05	2.17
**832**	U1334B	25.86	48	1.80	26.67	1.64	1.04	3.06
**833**	U1334B	25.86	52	1.93	26.98	1.64	1.03	3.27
**834**	U1334B	25.86	39	1.52	25.67	1.64	1.05	2.62
**835**	U1334B	25.86	63	2.11	29.86	1.64	1.05	3.65
**836**	U1334B	25.86	48	1.55	30.95	1.64	1.06	2.70
**837**	U1334B	25.86	47	1.48	31.83	1.64	1.09	2.64
**838**	U1334B	25.86	61	2.04	29.87	1.64	1.02	3.43
**839**	U1334B	25.86	32	1.06	30.27	1.64	1.02	1.77
**840**	U1334B	25.86	36	1.31	27.42	1.64	1.03	2.23
**841**	U1334B	25.86	29	0.99	29.27	1.64	1.03	1.68
**842**	U1334B	25.86	32	1.16	27.5	1.64	1.08	2.06
**843**	U1334B	25.86	39	1.31	29.72	1.64	1.07	2.32
**844**	U1334B	25.86	36	1.30	27.78	1.64	1.10	2.34

**845**	U1334B	25.86	22	0.77	28.62	1.64	1.06	1.33
**846**	U1334B	25.86	45	1.65	27.22	1.64	1.09	2.97
**847**	U1334B	25.86	20	0.70	28.75	1.64	1.09	1.25
**848**	U1334B	25.86	33	1.08	30.43	1.64	1.08	1.92
**849**	U1334B	25.86	27	0.87	31.06	1.64	1.06	1.52
**850**	U1334B	25.86	62	2.20	28.22	1.64	1.08	3.91
**851**	U1334B	25.86	17	0.60	28.48	1.64	1.07	1.05
**852**	U1334B	25.86	44	1.59	27.66	1.64	1.08	2.83
**853**	U1334B	25.86	29	0.96	30.11	1.64	1.07	1.70
**854**	U1334B	25.86	40	1.52	26.33	1.64	1.11	2.76
**855**	U1334B	25.86	24	0.91	26.48	1.64	1.07	1.59
**856**	U1334B	25.86	43	1.53	28.11	1.64	1.07	2.70
**857**	U1334B	25.86	18	0.63	28.54	1.64	1.07	1.11
**858**	U1334B	25.86	31	1.20	25.9	2.51	1.09	3.29
**859**	U1334B	25.86	16	0.69	23.29	2.51	1.08	1.86
**860**	U1334B	25.86	27	1.12	24.02	2.51	1.04	2.95
**861**	U1334B	25.86	38	1.42	26.69	2.51	1.04	3.73
**862**	U1334B	25.86	20	0.73	27.27	2.51	1.06	1.95
**863**	U1334B	25.86	32	1.17	27.28	2.51	1.06	3.12
**864**	U1334B	25.86	33	1.29	25.62	2.51	1.07	3.47
**865**	U1334B	25.86	32	1.24	25.77	2.51	1.08	3.38
**866**	U1334B	25.86	40	1.73	23.14	2.51	1.06	4.62
**867**	U1334B	25.86	23	1.00	23.07	2.51	1.07	2.67
**868**	U1334B	25.86	22	0.91	24.17	2.51	1.08	2.48
**869**	U1334B	25.86	13	0.61	21.33	2.51	1.03	1.58
**870**	U1334B	25.86	23	0.86	26.81	2.51	1.04	2.25
**871**	U1334B	25.86	14	0.47	30.02	2.51	1.07	1.26
**872**	U1334B	25.86	25	0.85	29.33	2.51	1.07	2.29
**873**	U1334B	25.86	30	1.21	24.75	2.51	1.04	3.17
**874**	U1334B	25.86	24	0.93	25.82	2.51	1.07	2.51
**875**	U1334B	25.86	19	0.69	27.41	2.51	1.05	1.83
**876**	U1334B	25.86	36	1.23	29.19	2.51	1.04	3.23
**877**	U1334B	25.86	26	0.85	30.5	2.51	1.08	2.31
**878**	U1334B	25.86	30	0.99	30.34	2.51	1.08	2.69
**879**	U1334B	25.86	28	0.91	30.89	2.51	1.10	2.50
**880**	U1334B	25.86	30	1.04	28.76	2.51	1.11	2.90
**881**	U1334B	25.86	23	0.94	24.58	2.51	1.12	2.63
**882**	U1334B	25.86	29	1.09	26.5	2.51	1.12	3.09
**883**	U1334B	25.86	25	0.92	27.25	2.51	1.12	2.58
**884**	U1334B	25.86	29	1.05	27.68	2.51	1.08	2.86
**885**	U1334B	25.86	21	0.79	26.75	2.51	1.10	2.17
**886**	U1334B	25.86	21	0.87	24.02	2.51	1.09	2.40
**887**	U1334B	25.86	22	0.90	24.56	2.51	1.11	2.51
**888**	U1334B	25.86	12	0.55	21.73	2.51	1.07	1.48
**889**	U1334B	25.86	19	0.85	22.25	2.51	1.09	2.34
**890**	U1334B	25.86	20	0.79	25.33	2.51	1.08	2.14
**891**	U1334B	25.86	33	1.21	27.21	2.51	1.07	3.28

**892**	U1334B	25.86	32	1.28	25.03	2.51	1.09	3.50
**893**	U1334B	25.86	28	1.11	25.31	2.51	1.11	3.09
**894**	U1334B	25.86	53	1.87	28.4	2.51	1.12	5.23
**895**	U1334B	25.86	37	1.58	23.43	2.51	1.09	4.34
**896**	U1334B	25.86	18	0.72	25.04	2.51	1.07	1.94
**897**	U1334B	25.86	23	1.00	23.1	1.53	1.03	1.56
**898**	U1334B	25.86	18	0.77	23.44	1.53	1.07	1.26
**899**	U1334B	25.86	19	0.75	25.4	1.53	1.06	1.21
**900**	U1334B	25.86	22	0.89	24.7	1.53	1.06	1.44
**901**	U1334C	25.86	17	0.90	18.88	1.53	1.05	1.44
**902**	U1334C	25.86	20	1.05	19.11	1.53	1.03	1.64
**903**	U1334C	25.86	10	0.53	18.79	1.53	1.04	0.84
**904**	U1334C	25.86	15	0.68	22.18	1.53	1.03	1.06
**905**	U1334C	25.86	17	0.68	24.97	1.53	1.05	1.09
**906**	U1334C	25.86	13	0.63	20.54	1.53	1.06	1.02
**907**	U1334C	25.86	11	0.50	22.06	1.53	1.04	0.79
**908**	U1334C	25.86	13	0.69	18.9	1.53	1.02	1.07
**909**	U1334C	25.86	6	0.33	18.07	1.53	1.01	0.51
**910**	U1334C	25.86	19	0.99	19.28	1.53	1.02	1.54
**911**	U1334C	25.86	19	0.95	20.02	1.53	1.06	1.54
**912**	U1334C	25.86	14	0.71	19.84	1.53	1.06	1.14
**913**	U1334C	25.86	32	1.03	31.03	1.53	1.09	1.72
**914**	U1334C	25.86	14	0.53	26.18	1.53	0.99	0.81
**915**	U1334C	25.86	11	0.35	31.36	1.53	1.02	0.55
**916**	U1334C	25.86	11	0.46	24.1	1.53	0.99	0.69
**917**	U1334C	25.86	30	1.33	22.54	1.53	0.95	1.94
**918**	U1334C	25.86	27	1.17	23.15	1.53	0.98	1.75
**919**	U1334C	25.86	14	0.56	25.18	1.53	0.99	0.84
**920**	U1334C	25.86	32	1.23	25.97	1.53	1.04	1.95
**921**	U1334C	25.86	30	1.13	26.44	1.53	1.04	1.81
**922**	U1334C	25.86	21	0.86	24.37	1.53	1.01	1.34
**923**	U1334C	25.86	46	1.96	23.44	1.53	1.03	3.09
**924**	U1334C	25.86	30	1.24	24.23	1.53	1.06	2.00
**925**	U1334C	25.86	35	1.60	21.88	1.53	1.02	2.50
**926**	U1334C	25.86	69	2.50	27.62	1.53	1.03	3.94
**927**	U1334C	25.86	24	1.11	21.56	1.53	1.05	1.78
**928**	U1334C	25.86	27	1.09	24.72	1.53	1.06	1.77
**929**	U1334C	25.86	36	1.51	23.85	1.53	1.07	2.48
**930**	U1334C	25.86	45	1.72	26.12	1.79	1.04	3.20
**931**	U1334C	25.86	46	2.08	22.15	1.79	1.06	3.91
**932**	U1334C	25.86	29	1.16	24.96	1.79	1.06	2.21
**933**	U1334C	25.86	34	1.29	26.44	1.79	1.04	2.39
**934**	U1334C	25.86	29	1.20	24.09	1.79	1.04	2.23
**935**	U1334C	25.86	25	1.09	22.92	1.79	1.05	2.03
**936**	U1334C	25.86	48	1.76	27.23	1.79	1.02	3.22
**937**	U1334C	25.86	18	0.64	28.27	1.79	0.96	1.10
**938**	U1334C	25.86	29	0.93	31.12	1.79	1.06	1.76

**939**	U1334C	25.86	25	1.32	18.93	1.79	1.04	2.44
**940**	U1334C	25.86	51	2.88	17.68	1.79	0.99	5.12
**941**	U1334C	25.86	21	1.04	20.19	1.79	1.05	1.95
**942**	U1334C	25.86	27	1.32	20.38	1.79	1.01	2.38
**943**	U1334C	25.86	23	0.94	24.55	1.79	1.00	1.68
**944**	U1334C	25.86	27	1.05	25.62	1.79	1.01	1.90
**945**	U1334C	25.86	25	0.91	27.48	1.79	0.99	1.62
**946**	U1334C	25.86	26	1.11	23.38	1.79	1.02	2.03
**947**	U1334C	25.86	26	1.13	22.93	1.79	0.99	2.01
**948**	U1334C	25.86	31	1.24	24.92	1.79	1.00	2.22
**949**	U1334C	25.86	33	1.24	26.61	1.79	1.01	2.23
**950**	U1334C	25.86	36	1.25	28.79	1.79	0.98	2.19
**951**	U1334C	25.86	19	0.90	21.2	1.79	0.90	1.44
**952**	U1334C	25.86	64	2.33	27.44	1.79	0.90	3.74
**953**	U1334C	25.86	19	0.88	21.57	1.79	0.96	1.51
**954**	U1334C	25.86	25	0.93	26.76	1.79	0.99	1.65
**955**	U1334C	25.86	29	0.90	32.3	1.79	0.99	1.59
**956**	U1334C	25.86	30	1.10	27.25	1.79	1.01	1.99
**957**	U1334C	25.86	42	1.41	29.85	1.79	0.97	2.44
**958**	U1334C	25.86	37	1.31	28.28	1.79	0.94	2.20
**959**	U1334C	25.86	42	1.53	27.5	1.79	0.97	2.65
**960**	U1334C	25.86	39	1.71	22.83	1.79	1.01	3.07
**961**	U1334C	25.86	40	1.77	22.66	1.79	1.05	3.29
**962**	U1334C	25.86	42	1.49	28.23	1.79	1.09	2.90
**963**	U1334C	25.86	33	1.10	30.06	1.79	1.08	2.11
**964**	U1334C	25.86	121	4.13	29.32	1.79	1.06	7.84
**965**	U1334C	25.86	40	1.64	24.46	1.79	1.09	3.19
**966**	U1334C	25.86	18	0.72	25.11	1.79	1.08	1.39
**967**	U1334C	25.86	35	1.43	24.42	1.79	1.12	2.87
**968**	U1334C	25.86	57	1.92	29.7	1.79	1.13	3.88
**969**	U1334C	25.86	68	2.54	26.82	1.75	1.15	5.08
**970**	U1334C	25.86	62	2.23	27.86	1.75	1.12	4.36
**971**	U1334C	25.86	50	2.23	22.45	1.75	1.08	4.19
**972**	U1334C	25.86	27	1.02	26.58	1.75	1.09	1.93
**973**	U1334C	25.86	41	1.25	32.73	1.75	1.12	2.44
**974**	U1334C	25.86	48	1.55	30.9	1.75	1.06	2.88
**975**	U1334C	25.86	47	1.71	27.49	1.75	1.03	3.08
**976**	U1334C	25.86	49	1.67	29.28	1.75	1.05	3.08
**977**	U1334C	25.86	56	2.22	25.19	1.75	1.04	4.03
**978**	U1334C	25.86	31	1.42	21.88	1.75	1.04	2.58
**979**	U1334C	25.86	11	0.50	21.92	1.75	1.02	0.89
**980**	U1334C	25.86	21	0.69	30.46	1.75	1.04	1.25
**981**	U1334C	25.86	21	0.68	30.71	1.75	1.01	1.20

LSR: linear sedimentation rate
DBD: dry bulk density
ASAR: atelostomate spine accumulation rate
