# Supplementary material for: A 104-Ma record of deep-sea Atelostomata (Holasterioda, Spatangoida, irregular echinoids) – a story of persistence, food availability and a big bang
Source: PLoS One. 2023 Aug 9;18(8):e0288046. doi: 10.1371/journal.pone.0288046 (PMC10411753; doi:10.1371/journal.pone.0288046)
Supplement: S6 Table — (PDF) [file pone.0288046.s006.pdf]

data set Holes U1406A, B

sample id	hole	core	interval	age in Fig. 7	spines	spines/g	dry weight (g)	LSR	DBD
1	U1406B	12H-5W	82-84	25.10	34	1.61	21.07	1.37	0.73
2	U1406A	12H-3W	118-120	25.10	18	0.88	20.47	1.37	0.80
3	U1406B	13H-2W	130-132	25.10	5	0.25	20.34	2.22	0.82
4	U1406B	13H-3W	118-120	25.10	7	0.35	20.22	2.22	0.82
5	U1406B	13H-4W	22-24	25.10	9	0.45	20.08	2.22	0.82
6	U1406B	13H-6W	22-24	25.10	3	0.19	15.46	2.22	0.82
7	U1406A	13H-3W	36-38	25.10	6	0.34	17.48	2.22	0.82
8	U1406A	13H-3W	4-6	25.10	5	0.47	10.58	2.22	0.74
9	U1406A	13H-3W	26-28	25.10	9	0.55	16.41	2.22	0.74

LSR: linear sedimentation rate
DBD: dry bulk density
ASAR: atelostomate spine accumulatior

ASAR
1.61
0.96
0.45
0.63
0.82
0.35
0.62
0.78
0.90

ı rate
