# Supplementary material for: A 104-Ma record of deep-sea Atelostomata (Holasterioda, Spatangoida, irregular echinoids) – a story of persistence, food availability and a big bang
Source: PLoS One. 2023 Aug 9;18(8):e0288046. doi: 10.1371/journal.pone.0288046 (PMC10411753; doi:10.1371/journal.pone.0288046)
Supplement: S7 Table — (PDF) [file pone.0288046.s007.pdf]

data set Holes U1407A, C

sample id	hole	core	age in Fig. 7	spines	spines/g	dry weight (g)	LSR	DBD	ASAR
**post- OAE2**									
1	U1407A	27H-1X	93.00	15	1.00	15.00	0.05	1.21	0.06
2	U1407A	27H-1X	93.00	14	1.39	10.10	0.05	1.21	0.08
3	U1407A	27H-1X	93.00	10	0.93	10.80	0.05	1.21	0.06
4	U1407A	27H-3X	93.00	52	3.56	14.60	0.86	1.15	3.52
5	U1407A	27H-3X	93.00	8	0.68	11.80	0.86	1.15	0.67
6	U1407A	27H-3X	93.00	17	1.24	13.70	0.86	1.15	1.23
7	U1407A	27H-3X	93.00	6	0.47	12.70	0.86	1.15	0.47
8	U1407A	27H-4X	93.00	2	0.13	15.30	0.86	1.15	0.13
9	U1407A	27H-4X	93.00	13	0.54	24.10	0.86	1.15	0.53
10	U1407A	27H-4X	93.00	30	2.29	13.10	0.86	1.15	2.26
11	U1407A	27H-4X	93.00	15	0.90	16.60	0.86	1.15	0.89
12	U1407A	27H-4X	93.00	13	0.76	17.10	0.86	1.15	0.75
13	U1407A	27H-4X	93.00	1	0.06	16.50	0.86	1.15	0.06
14	U1407A	27H-4X	93.00	11	0.52	21.00	0.86	1.15	0.52
15	U1407A	27H-5X	93.00	3	0.17	17.50	0.86	1.15	0.17
16	U1407A	27H-5X	93.00	10	0.48	20.80	0.86	1.15	0.48
17	U1407A	27H-5X	93.00	3	0.12	24.30	0.86	1.15	0.12
18	U1407A	27H-5X	93.00	12	0.71	16.80	0.86	1.15	0.71
19	U1407A	27H-5X	93.00	37	1.91	19.40	0.86	1.15	1.89
20	U1407A	27H-5X	93.00	10	0.69	14.40	0.86	1.15	0.69
21	U1407A	27H-6X	93.00	13	0.62	21.00	0.86	1.15	0.61
22	U1407A	27H-6X	93.00	10	0.39	25.70	0.86	1.15	0.38
23	U1407A	27H-6X	93.00	3	0.15	20.00	0.86	1.15	0.15
**pre-OAE2**									
24	U1407C	26H-5X	94.00	73	4.80	15.20	0.39	1.47	2.78
25	U1407C	26H-5X	94.00	29	2.50	11.60	0.39	1.47	1.45
26	U1407C	26H-6X	94.00	25	2.34	10.70	0.39	1.47	1.35
27	U1407C	26H-6X	94.00	40	3.45	11.60	0.39	1.47	2.00
28	U1407C	26H-6X	94.00	3	0.29	10.20	0.39	1.47	0.17
29	U1407C	26H-6X	94.00	30	2.31	13.00	0.39	1.47	1.34
30	U1407C	26H-6X	94.00	19	1.78	10.70	0.39	1.47	1.03
31	U1407C	26H-6X	94.00	9	0.55	16.30	0.39	1.47	0.32
32	U1407C	26H-6X	94.00	9	0.74	12.20	0.39	1.47	0.43
33	U1407C	26H-6X	94.00	5	0.33	15.00	0.39	1.47	0.19
34	U1407C	27H-1X	94.00	30	2.36	12.70	0.39	1.47	1.37
35	U1407C	27H-1X	94.00	14	0.79	17.80	0.39	1.47	0.46
36	U1407C	27H-1X	94.00	123	5.17	23.80	0.39	1.47	2.99
37	U1407C	27H-2X	94.00	41	1.88	21.80	0.39	1.47	1.09
38	U1407C	27H-3X	94.00	63	2.94	21.40	0.39	1.47	1.71
39	U1407C	27H-3X	94.00	53	2.79	19.00	0.39	1.47	1.62
40	U1407C	27H-4X	94.00	76	3.60	21.10	0.39	1.47	2.09
41	U1407C	27H-5X	94.00	10	0.48	21.00	0.39	1.47	0.28
42	U1407C	27H-6X	94.00	4	0.19	21.10	0.39	1.47	0.11

LSR: linear sedimentation rate
DBD: dry bulk density
ASAR: atelostomate spine accumulation rate
